# Predicting the sensitivity and specificity of published real-time PCR assays

**DOI:** 10.1186/1476-0711-7-18

**Published:** 2008-09-25

**Authors:** Gordon H Lemmon, Shea N Gardner

**Affiliations:** 1 Center for Structural Biology, Vanderbilt University, 465 21st Ave. South, BIOSCI/MRB III suite 5140, Nashville, TN 37240, USA; 2 Pathogen Bioinformatics, Lawrence Livermore National Laboratory, PO Box 808, L-174, Livermore, CA 94551, USA

## Abstract

**Background:**

In recent years real-time PCR has become a leading technique for nucleic acid detection and quantification. These assays have the potential to greatly enhance efficiency in the clinical laboratory. Choice of primer and probe sequences is critical for accurate diagnosis in the clinic, yet current primer/probe signature design strategies are limited, and signature evaluation methods are lacking.

**Methods:**

We assessed the quality of a signature by predicting the number of true positive, false positive and false negative hits against all available public sequence data. We found real-time PCR signatures described in recent literature and used a BLAST search based approach to collect all hits to the primer-probe combinations that should be amplified by real-time PCR chemistry. We then compared our hits with the sequences in the NCBI taxonomy tree that the signature was designed to detect.

**Results:**

We found that many published signatures have high specificity (almost no false positives) but low sensitivity (high false negative rate). Where high sensitivity is needed, we offer a revised methodology for signature design which may designate that multiple signatures are required to detect all sequenced strains. We use this methodology to produce new signatures that are predicted to have higher sensitivity and specificity.

**Conclusion:**

We show that current methods for real-time PCR assay design have unacceptably low sensitivities for most clinical applications. Additionally, as new sequence data becomes available, old assays must be reassessed and redesigned. A standard protocol for both generating and assessing the quality of these assays is therefore of great value. Real-time PCR has the capacity to greatly improve clinical diagnostics. The improved assay design and evaluation methods presented herein will expedite adoption of this technique in the clinical lab.

## Background

Real-time PCR assays are gaining popularity as a clinical tool for detecting and quantifying the presence of both viral and bacterial pathogens, as reviewed in [1]. Compared to traditional culturing methods used in identification, real-time PCR is fast and cost effective. In addition, it can be quantitative and sensitive, in some cases greatly exceeding the sensitivity for conventional testing methods. Commercially distributed kits are available for PCR-based pathogen diagnostics, and PCR is no longer thought of merely as confirmatory to culture. However real-time PCR assays are limited by the quality of the primers and probes chosen. These primers and probes must be sensitive enough to match all target organisms yet specific enough to exclude all others.

A common approach to developing a primer/probe combination is by using commercial software such as PrimerExpress^® ^(Applied Biosystems, Foster City, CA, USA). This software asks the user to upload a DNA sequence file, and then finds possible primer/probe sets that meet the assay criteria. Generally a researcher will provide as input a gene region conserved throughout the taxa that the assay is being designed to detect. The software then provides possible primer/probe sets. The researcher chooses a representative signature. If there are single nucleotide polymorphisms (SNPs) within the chosen conserved region, a signature with consensus primers and probes is often chosen. Next a BLAST [2] search is performed to ensure that the primers are not hitting other targets. Finally the signature is verified *in vitro *with laboratory strains.

While this design approach may work acceptably well in the research laboratory, the clinical laboratory calls for a more thorough analysis to ensure detection of novel, diverse, and uncommon strains. These may appear, for example, as a result of spread by foreign travel or migration. Whole genome based automated signature design [3] presents a great improvement to the common method. However, in addition to better design strategies, methods for automated signature evaluation are needed. As additional sequence data becomes available, it is necessary to regularly reassess the predicted efficacy of a given signature. This analysis must include the predicted false negative and false positive rates for the developed signatures, and consider all available public sequence data.

We have analyzed a number of real-time PCR assays found in the literature based on public sequence data. Herein we report how well these signatures performed, offer a revised approach to PCR assay design, and use this approach to produce new assays predicted to have higher sensitivity and specificity.

## Methods

### Literature search

The literature was combed for recently published articles reporting real-time PCR assays for the clinical detection of bacterial and viral taxa. The primer and probe sequences were accumulated, with a preference for TaqMan assays. However, 3 intercalating dye assays were also selected. Papers reporting nucleotide sequences that could not easily be copied from an online source were avoided. In total, 112 signatures from 32 papers were analyzed.

### Database construction

Local Oracle databases have been constructed from the complete genome sequence data available at NCBI Genbank, TIGR, EMBL, IMG (JGI), and Baylor HGSC. We used our "all_virus" and "all_bacteria" databases to find signature matches and predict false negatives and false positives. These databases were designed to contain only whole genomes and whole segments from segmented genomes. However, the heuristics used to separate whole genomes from partial sequences are not fail-proof due to inconsistency in sequence annotation within the public databases. Consequently many sequences in these databases may show up as false negatives when they are actually just a section or segment of a genome that is not expected to contain the signature, and we manually sorted these sequences into true or false negatives.

### BLAST search

A freely available real time PCR analysis tool called TaqSim [4] was used to find public sequences that would match the primer/probe assay in question. TaqSim uses BLAST searches to find sequences that match both forward and reverse primers and probe. To be reported as a "hit" the primers and probe must match in the required orientations relative to one another and the primers must be in sufficiently close proximity. The forward/reverse primers may fall on either the plus or minus strand, so long as the orientation relative to one another is appropriate. There may not be mismatches at the 3' end of either primer. For each hit TaqSim calculates the primer and probe melting temperatures as bound to the candidate hit sequence (accounting for mismatches) based on reaction conditions (reagent concentrations and hybridization temperature), and returns sequences predicted to be amplified. Instead of replicating the various exact reaction conditions reported in each paper, very lenient settings were applied in all cases, essentially removing the screen for primer/probe vs. candidate hit Tm by setting this threshold to 0°K, and instead checking for specificity by requiring that hits have fewer than 3 mismatches per primer or probe.

### Signature/taxonomy comparison

TaqSim's predicted sequence hits were compared with sequences listed under a given set of NCBI taxonomy tree nodes. For instance, if a signature was reported to detect Hepatitis B, then its set of TaqSim hits would be compared with the set of sequences under node 10407, corresponding to Hepatitis B virus. Sequences in both sets were considered true positives, sequences in the TaqSim output that were missing from the chosen taxonomy nodes were considered false positives, and sequences that were in the taxonomy tree but missing from the TaqSim output were considered false negatives. Test statistics such as specificity and sensitivity (power) were then calculated. In this paper we define sensitivity and specificity as follows:

$$Sensitivity=1-false\;negative\;rate=1-\frac{false\;negatives}{true\;positives+false\;negatives}$$

$$Specificity=1-false\;positive\;rate=1-\frac{false\;positives}{true\;negatives+false\;positives}$$

### Taxonomy node selection

The primary research articles were read carefully to determine what the authors had designed their primers/probes to detect. NCBI Taxonomy nodes were chosen to represent these target organisms. This was not a trivial task, since many articles lack clarity as to which taxa, specifically, their assay should detect. For instance, the cytomegalovirus assay did not detect all sequences in the cytomegalovirus genus (taxonomy node 10358), but rather all sequences in the human herpesvirus 5 species (taxonomy node 10359). None of the articles specified a taxonomy node for their signatures.

### Hand curating

Perl scripting [5] was used to help compare BLAST hits and taxonomy node sequences, and count false negative, false positive and true positive sequence matches. However some sequences required hand sorting due to the wide array of sequence types and annotations. These often represented segmented genomes, in which case many of the would-be false negative sequences simply represent a different segment than that on which the signature lands, so we manually tabulated them as true negatives. They may also represent plasmids. In these situations, a careful review of the Genbank entry, and sometimes of the primary article cited by Genbank, was necessary to determine if the sequence of interest was truly a false negative.

Although we attempt to include only complete genomes in our sequence database, because of inconsistencies in the annotation of sequence data some partial sequences nevertheless make it into our databases. Any of these partial CDS's documented as containing the target gene on which the signature was supposed to land were counted as false negatives, but those partial CDS's not documented to contain the target gene were eliminated from the false negative pool because it is possible the signature could land on the unsequenced section with the target gene. Our database also contains "glued fragments", which represent draft genomes "glued" together with hundreds of "N"s as a simple way to keep the separate contigs associated as part of the same genome. While we report false negatives from these draft genomes, it is possible that the signatures could land on gaps between the contigs, and that finished sequencing could result in re-classification as a true positive.

## Results

Tables 1, 2, 3, 4, 5 summarize our analysis of various DNA signatures. Details of all true positive, false positive and false negative sequences are available from the authors. Note that these results are *in silico *results; no laboratory testing was performed for verification, so that by stating that an organism is "detected" we mean that this is our prediction based on sequence data. A few notes of interest concerning the data in the tables are described below:

**Table 1:** viral signature analyses.

**Reference Number**	***Scope***	**Year**	**Target Organism/gene**	**True Positives**	**False Positives**	**False Negatives**	**Sensitivity**
[6]	Strain	2004	Human coronavirus OC43	5	17	0	1.00
[6]	Strain	2004	Human coronavirus 229E	1	0	0	1.00
[7]	Strain	2004	Coxsackie virus B3	8	0	2	0.80
[8]	Strain	2004	Coxsackie virus B4	3	78	0	1.00
[20]	Serotype	2007	Influenza H5 strains	26	0	402	0.06
[21]	Species	2004	Influenza A MP segment	238	0	3726	0.06
[21]	Species	2004	Influenza B HA segment	131	0	238	0.36
[10]	Species	2002	Dengue virus	0	0	185	0.00
[16]	Serotype	2006	Dengue virus type 1	0	0	47	0.00
[16]	Serotype	2006	Dengue virus type 2	0	0	57	0.00
[16]	Serotype	2006	Dengue virus type 3	2	0	68	0.03
[16]	Serotype	2006	Dengue virus type 4	0	0	11	0.00
[21]	Species	2004	Adenovirus A	1	0	0	1.00
[21]	Species	2004	Adenovirus B	17	0	3	0.85
[21]	Species	2004	Adenovirus C	6	0	0	1.00
[21]	Species	2004	Adenovirus D	6	0	0	1.00
[21]	Species	2004	Adenovirus E	12	0	0	1.00
[21]	Species	2004	Adenovirus F	2	0	0	1.00
[10]	**Species**	**2002**	**Lassa Virus**	**1**	**0**	**14**	**0.08**
[9]	Species	2004	Ebola Sudan	1	0	0	1.00
[9]	Species	2004	Ebola Zaire	5	0	0	1.00
[9]	Species	2004	Marburg Virus	13	0	5	0.72
[22]	Species	1996	Hepatitis C virus	102	0	33	0.76
[10]	Species	2002	Rift Valley fever virus	36	0	5	0.88
[23]	Species	2000	West Nile Virus 3'NC	71	0	13	0.85
[23]	Species	2000	West Nile Virus- ENV	71	0	13	0.85
[15]	Species	2007	West Nile virus- RdRp	49	0	35	0.58
[15]	Species	2007	Japanese encephalitis virus	0	0	43	0.00
[15]	Species	2007	Yellow fever virus	0	0	15	0.00
[15]	Species	2007	St. Louis encephalitis virus	0	0	2	0.00
[10]	Species	2002	Yellow fever virus	10	0	5	0.67
[24]	Species	2000	Hepatitis B virus (1)	942	0	56	0.94
[25]	Species	2000	Hepatitis B virus (2)	641	0	357	0.64
[26]	Species	2004	Cytomegalovirus	4	0	3	0.57
[21]	Species	2004	Cytomegalovirus	4	0	3	0.57
[21]	Species	2004	Epstein-Barr (HHV 4)	4	0	0	1.00
[27]	Species	2005	Herpes Simplex Virus 1	1	0	0	1.00
[21]	Species	2004	Herpes Simplex Virus 1	1	0	0	1.00
[27]	Species	2005	Herpes Simplex Virus 2	1	0	0	1.00
[21]	Species	2004	Herpes Simplex Virus 2	1	0	0	1.00
[28]	Species	1999	Varicella-Zoster	19	0	0	1.00
[21]	Species	2004	Varicella-Zoster	19	0	0	1.00
[21]	Species	2004	Human herpesvirus 6	3	0	0	1.00
[21]	Species	2004	Human herpesvirus 7	1	0	0	1.00
[21]	Species	2004	Human herpesvirus 8	2	0	0	1.00
[29]	Species	2005	Mumps virus	12	0	5	0.71
[30]	Species	2004	Newcastle Disease irus	23	0	15	0.61
[21]	Species	2004	Parainfluenzaviruses-1	1	0	0	1.00
[21]	Species	2004	Parainfluenzaviruses-2	4	0	1	0.80
[21]	Species	2004	Parainfluenzaviruses-3	2	0	0	1.00
[21]	Species	2004	Respiratory syncytial virus	7	0	3	0.70
[21]	Species	2004	Human parvovirus B19	3	0	0	1.00
[21]	Genus	2004	Enteroviruses	170	0	21	0.89
[21]	Species	2004	JC polyomavirus	378	0	2	1.00
[21]	Species	2004	BK polyomavirus	120	378	0	1.00
[31]	Species	2005	HIV – type 1	283	1	820	0.26
[10]	**Species**	**2002**	**Crimean-Congo hemorrhagic fever**	**27**	**0**	**21**	**0.56**
[32]	Genus	2001	Enterovirus genus	171	0	19	0.90
[10]	**Family**	**2002**	**Ebola and Marburg**	**6**	**0**	**21**	**0.22**

All but three are Taqman signatures. The three intercalating dye type assays are bolded.

**Table 2:** assorted bacterial DNA signature analyses.

	***Scope***	**Year**	**Target Organism/gene**	**True Positives**	**False Positives**	**False Negatives**	**Sensitivity**
[33]	Species	2004	Neisseria gonorrhoeae1	1	0	0	1.00
[33]	Species	2004	Neisseria gonorrhoeae2	1	0	0	1.00
[34]	Species	2003	Chlamydia pneumonia	4	0	0	1.00
[35]	Species	2007	Borrelia plasmid Ip54	2	0	0	1.00
[12]	Genus	2004	Bacteroides	0	0	9	0.00
[12]	Species	2004	Escherichia coli	25	11	5	0.83
[36]	Species	2005	Ehrlichia chaffeensis	1	0	0	1.00
[12]	Species	2004	Ehrlichia canis	1	0	0	1.00
[11]	Species	2000	Staphylococcus aureaus	9	2	4	0.69
[12]	Species	2004	Haemophilus influenzae	0	0	15	0.00
[12]	Species	2004	Pseudomonas aeruginosa	0	0	7	0.00
[12]	Genus	2004	Acinetobacter spp.	2	0	0	1.00
[12]	Family	2004	Enterobacteriaceae	45	0	42	0.52
[12]	Species	2004	Stenotrophomonas maltophilia	0	0	1	0.00

**Table 3:** Analysis of measles virus assays from Hummel, 2006 [13].

***Scope***	**Target Gene**	**True Positives**	**False Positives**	**False Negatives**	**Sensitivity**
Species	Measles F1	15	0	2	0.88
Species	Measles N1	16	0	1	0.94
Species	Measles F2	15	0	2	0.88
Species	Measles F4b	16	0	1	0.94
Species	Measles F3	9	0	8	0.53
Species	Measles N2	10	0	7	0.59
Species	Measles N3	17	0	0	1.00
Species	Measles H1	10	0	7	0.59
Species	Measles H2	16	0	1	0.94
Species	Measles H3	9	0	8	0.53
Species	Measles H4b	14	0	3	0.82
Species	Measles N4b	11	0	6	0.65

**Table 4:** Analysis of hepatitis assays from Gardner et al, 2003 [14].

***Scope***	**Target Organism**	**True Positives**	**False Positives**	**False Negatives**	**Sensitivity**
Species	Hepatitis A-1	14	0	3	0.82
Species	Hepatitis A-2	14	0	3	0.82
**Species**	**All Hepatitis As**	**14**	**0**	**3**	**0.82**
Species	Hepatitis B-1	927	2	37	0.96
Species	Hepatitis B-2	799	0	165	0.83
**Species**	**All Hepatitis Bs**	**959**	**2**	**5**	**1.00**
Species	Hepatitis C-1	130	0	4	0.97
Species	Hepatitis C-2	76	0	58	0.57
**Species**	**All Hepatitis Cs**	**133**	**0**	**1**	**0.99**
Species	Hepatitis E-1	25	0	40	0.39
Species	Hepatitis E-2	11	0	54	0.17
Species	Hepatitis E-3	20	0	45	0.31
**Species**	**All Hepatitis Es**	**55**	**0**	**10**	**0.85**

Clustered signature results are bolded.

**Table 5:** Signatures predicted not to hit any target organisms

	**Target Organism**	**Year**	**Analysis**
[15]	Japanese encephalitis virus	2007	Too many mismatches in either forward or reverse primer. Several strains have 3 mismatches at 3' end of forward primer in addition to internal mismatches.
[15]	Yellow fever virus	2007	Reverse primer only has a blast hit to one strain (Angola71). Forward primer only has blast hits to 3 strains, and there are many mismatches (e.g. for Angola71, the 11 bases at the 3' end of the primer do not match).
[15]	Saint Louis encephalitis virus	2007	Too many mismatches in the reverse primer, with 3 mismatches at 3' end as well as at other locations.
[16]	Dengue virus 1	2006	Reverse primer does not have any BLAST hits to target.
[16]	Dengue virus 2	2006	Forward primer has 3 or 12 mismatches at 3' end for most strains, the probe has BLAST hits to only 7 of the 57 genomes available, and reverse primer only has a BLAST hit to 1 genome but there are 3 mismatches at the 3' end.
[16]	Dengue virus 4	2006	Too many mismatches in forward primer and in some cases the probe.
[10]	Dengue virus	2002	Too many mismatches in forward primer. However, they are at the 5' end, so assay could still work for some strains with 19 matches at the 3' end of the forward primer.
[12]	Pseudomonas aeruginosa	2004	No blast hits of probe to Pseudomonas aeruginosa
[12]	Bacteroides spp.	2004	Probe is not between or even in close proximity to the forward and reverse primers
[12]	Stenotrophomonas maltophilia	2004	No blast hits of probe to Stenotrophomonas maltophilia
[12]	Haemophilus influenzae	2004	Probe only matches in 17 of 22 bases, which is unlikely to give a strong signal, since probe is unlikely to bind prior to the primers as desired for real time TaqMan chemistry.

### Assorted viral signatures (table 1)

The two human corona virus strains, 229E and OC43 are a frequent cause of the common cold [6]. A Taqman assay for 229E was predicted to perform perfectly, while an assay for OC43 turned up a number of false positives, all of which were animal corona viruses. A coxsackie B3 virus assay [7] performed well, but a coxsackie B4 assay [8] hit many other human coxsackie, echo, and entero viruses. Four out of 5 false negatives for a Marburg virus assay [9] were of the Lake Victoria variety. False negatives associated with a yellow fever signature [10] included Trinidad, French neurotropic, French viscerotropic, and vaccine strains. The Filoviridae (Ebola/Marburg) assay [10] detected only Ebola viruses.

### Assorted bacterial signatures (table 2)

Staphylococcus aureus [11] and Enterobacteriaceae assays [12] had low sensitivity. An Escherichia coli assay [12] hit Shigella and Vibrio sequences.

### Measles signature set (table 3)

Many of these signatures [13] had high sensitivity. Combining several of them into a multiplex assay would probably improve sensitivity further.

### Hepatitis signature set (table 4)

These signatures were designed using a minimal set clustering approach [14]. While individual signatures have decent sensitivity, combining several signatures in one assay, as advocated in the publication greatly improved sensitivity. The signatures for Hepatitis A are currently undergoing laboratory screening by the FDA, and are performing well (G. Hartman, personal communication).

### Signatures that had no hits (table 5)

Several reported signatures produced no predicted hits. These include assays for several flaviviruses [10,15,16], and 16S rRNA assays [12] for several bacteria. Examination of BLAST output showed that in these cases either a primer or internal oligo (probe) did not have BLAST hits to target, there were too many mismatches per primer or probe sequence above the threshold specified in our analyses, or there were mismatches at the 3' end of a primer relative to target. It is possible that if the sequences of the samples used in the laboratory differ from available genomic data, or if the PCR reaction conditions are performed at low stringency (e.g. low annealing temperatures or high salt concentrations) these assays could in fact work in the laboratory. However, according to the genomic data available, a better match of primers and probes to target is possible and is usually desired for high sensitivity detection.

### Improved signatures using minimal set clustering (additional file)

Targeting a number of the organisms for which currently published signatures were predicted to perform poorly, as well as some for which additional signatures may be desired (even though published signatures may perform well), we generated new signatures using Minimal Set Clustering (MSC) according to methods previously described [14,17]. MSC begins by removing non-unique regions from consideration as primers or probes from each of the target sequences relative to a database of non-target bacterial and viral sequences. The remaining unique regions of each target sequence are mined for all or many candidate signatures, without regard for conservation among other targets, yet satisfying user specifications for primer and probe length, T_m_, GC%, avoidance of long homopolymer runs, and amplicon length. All candidate signatures are compared to all targets and clustered by the subset of targets they are predicted to detect. Signatures within a given cluster are equivalent, in that they are predicted to detect the same subset of targets, so by clustering we reduce the redundancy and size of the problem to finding a small set of signatures that detect all targets. Nevertheless, finding the optimal solution of the fewest clusters to detect all targets is an NP complete problem, so for large data sets we use a greedy algorithm to find a small number of clusters that together should pick up all targets. In cases where the target strains of a species are too diverse to be detected by a single signature that hits all target strains, the MSC software groups the targets into genetic clusters to enable signature generation per group. If there are single signatures that are sufficient to detect all targets, then each of those comprehensive signatures is reported. We have used this method to design signature sets for numerous viruses, including Influenza A HA serotypes, foot-and-mouth-disease virus, Norwalk, Crimean Congo hemorrhagic fever, Ebola, hepatitis A, and other divergent viruses. Many of these signatures have been tested by collaborators at Lawrence Livermore National Laboratory, US Centers for Disease Control (CDC), USDA, FDA, and elsewhere.

In the supplementary table, we often provide more than one alternative signature to detect a given equivalence group of genomes to serve as a backup should a signature perform poorly in laboratory testing. Some of the signatures may have mismatches to some of their intended targets, although these mismatches are not predicted to reduce the T_m _of primer/probe hybridizing to target below typical TaqMan reaction conditions. None of these computationally predicted signatures have been screened in the laboratory, as this is beyond the scope of this paper.

The supplementary table contains signatures for the following organisms: Viruses: Dengue 1–4, human adenovirus A-F, canine distemper, coxsackie B4, cytomegalovirus, human herpes 1–4, 6–7, Japanese encephalitis, mumps, newcastle disease, Sendai, St. Louis encephalitis, human papillomavirus type 16, 71, human parainfluenza 1–3, JC, BK, and WU polyomavirus, West Nile, yellow fever, rabies, human respiratory syncytial, influenza A-B, influenza A serotypes H1, H2, H3, and H5, and HIV-1; Bacteria: Haemophilus influenzae, Mycobacterium tuberculosis, Staphylococcus aureus, Ehrlichia (genus), E. chaffeensis, E. ruminantium, Chlamydia trachomatis, Pseudomonas aeruginosa, Escherichia coli, and Neisseria meningitidis.

## Discussion

As expected we found that false negatives were much more common than false positives. Though signatures are generally based on conserved gene regions, they often fail to take into account all of the variation within a target set of organisms. This may be because the signatures were developed using sequence data from a handful of strains, rather than a thorough study of all strains publicly available.

These false negatives may also represent sequences that have become available since the publication of the given signature. Since new sequence data is made available at an ever increasing rate, there is great benefit in re-evaluating clinically used DNA signatures regularly. When new sequence data leads to false negative predictions for a signature, one of two explanations can be given. The new sequences either represent recently recognized variation that has been around since the time the signature was published, or new variation, the result of mutation and natural selection. In either case, an improved or additional signature should be designed.

High false positive or false negative rates do not necessarily indicate a "bad" DNA assay. The quality of an assay must be considered in light of the milieu in which the testing will take place. In the clinical laboratory, a signature with high sensitivity but perhaps low specificity may be preferred over a test with lower sensitivity in cases where the putative pathogen requires immediate treatment or may spread quickly. The case of antibiotic resistant bacteria probably falls in this category. On the other hand, the nation's BASIS and BioWatch programs insists on zero false positives, so as to avoid public disturbances due to false alarms, while still aiming for zero false negatives [18].

One must also consider the type of false negative and false positive results to determine their relevance. For instance, in this article an assay for human corona virus OC43 [6] was reported with a number of false positives to animal corona viruses. However, in the clinical lab in the US, need the clinician worry about the possibility of a false positive match to giraffe coronavirus in human sputum? What about such a match in a clinical lab in Africa? On the other hand, the echovirus sequences that the coxsackie B4 assay [8] can detect could produce misleading results in any clinical lab.

The false negative and false positive rates presented in this study may vary substantially from those seen empirically. This is because the strains available in a laboratory may differ significantly from the sequence data available, or because the empirical protocol is more or less stringent than the sequence-based requirements we imposed, which allowed no more than 2 mismatches per primer or probe for detection.

We believe that as more target sequences become available, our predicted false negative rates will tend to increase for a given published signature both as a result of better sampling of diversity and as a result of failure to detect newly evolved variants. It has been estimated that a minimum of 3–4 genomes are needed in order to computationally design TaqMan PCR signatures likely to detect most strains, with those isolates chosen for sequencing that have been selected to span gradients of geographic, phenotypic, and temporal variation [19]. Even more than 4 genomes are needed for particularly diverse organisms. Thus, older signatures may not perform as well as newly developed signatures from the most up-to-date sequence data. A future study of interest would be a longitudinal look at how these rates continue to change over time as additional sequences become available. This study could be performed retrospectively, since sequence submission dates are easily obtained from public databases.

We also hypothesized that the wider the intended scope of a signature, the lower its sensitivity would tend to be. The point is illustrated loosely in our data tables. Twenty-six of the 28 signatures with less than 10 publicly available target sequences had sensitivities of 1 (i.e. zero false negatives), while signatures with 10 or more targets had an average sensitivity of 0.710116. However this approach only considers scope in the context of sequence data available.

**Figure 1:**
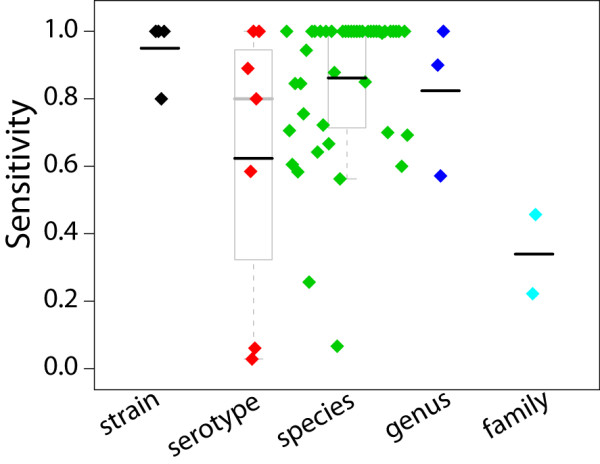
**Sensitivity by taxonomy level**. Each colored diamond represents a real-time PCR assay examined in this paper. Black bars indicate the mean, grey bars indicate the median. Top and bottom of each box indicates 75th and 25th percentiles, and grey lines at whisker ends denote min and max values. The wide ranging sensitivities demonstrate both inconsistency in genetic diversity at a given taxonomy level, and inconsistency in signature design approaches.

We tried to demonstrate the relationship between specificity and scope at a more fundamental level by grouping signatures by the taxonomic level of their target as shown in Figure 1. However the results are misleading. In virology, taxonomic level is not a good indicator of nucleotide diversity. For instance, there is more diversity in the influenza A *species *then there is in the entire *Filoviridae *family, which consists of only two known genera: Ebola-like viruses and Marburg viruses. A better approach might be to calculate nucleotide diversity as a function of phylogenetic branch length or shared k-mer clusters within a target taxonomy node.

Finally, we averaged the sensitivities of microbes by genome type as shown in Table 6. Note that the ssRNA-RT category includes only HIV-1. This chart demonstrates that creating signatures with high sensitivity becomes more difficult for target organisms with high mutation rates.

**Table 6:** sensitivity by type.

	***avg*.**	***std dev***
dsDNA	0.97	0.10
dsDNA-RT	0.79	0.21
Bacteria	0.86	0.86
ssRNA	0.71	0.28
ssRNA-RT	0.26	

Average sensitivities of bacteria and of single stranded (ss), and double stranded (ds) viruses, with and without reverse transcriptase (RT). Results are consistent with high mutation rates in groups for which signatures have lower sensitivity. ssRNA-RT has no standard deviation as it refers only to HIV.

## Conclusion

Current real-time PCR assay design approaches produce signatures with sensitivities generally too low for clinical use. We suggest that a rigorous approach involving false positive and false negative analysis should be the standard by which an initial assessment of signature quality is made. Signatures must also regularly be reassessed as sequence data becomes available. For targets with wide nucleotide diversity, it becomes necessary to develop a set of signatures, for which we suggest a minimal set clustering approach that may also include signatures with degenerate/inosine bases.

## Competing interests

The authors declare that they have no competing interests.

## Authors' contributions

GL found real time PCR signatures in the literature, wrote Perl scripts, and performed the analysis of published signatures. SG conceived of the research, designed new signatures, and provided guidance throughout the study.

## Supplementary Material

Additional file 1 **NewRealTimePCRSigatures**. Fifty Seven TaqMan PCR primer/probe combinations we predict to have higher sensitivity/specificity than current published assays. Click here for file
