# Supplementary material for: Predicting the sensitivity and specificity of published real-time PCR assays
Source: Ann Clin Microbiol Antimicrob. 2008 Sep 25;7:18. doi: 10.1186/1476-0711-7-18 (PMC2566554; doi:10.1186/1476-0711-7-18)
Supplement: Additional file 1 — NewRealTimePCRSigatures. Fifty Seven TaqMan PCR primer/probe combinations we predict to have higher sensitivity/specificity than current published assays. [file 1476-0711-7-18-S1.doc]

**Newly predicted signatures for a number of bacterial and viral pathogens.**

“Size of MSC soln” is the minimal (or near-minimal) number of signatures such each sequenced genome should be detected by one or more signatures, so that the set of signatures should in combination detect all the targets. Some additional signatures are provided which are not part of an MSC solution, as indicated by N, in cases where these signatures are predicted to detect a large fraction, but not all, of the target sequences. The predicted number of true positives is indicated. There were no false positives except for 2 of the Newcastle disease virus signatures, as indicated in the footnote. In some cases, more than one alternative signature is given that is predicted to detect the same set or subset of target sequences, as indicated by the same “group#” in the Signature ID for a given target organism (identical group#’s are followed by a unique signature # for a given organism). Different group#’s are predicted to detect different subsets of the target sequences. In some cases, alternative MSC solutions are provided. Amplicons in the predicted targets are provided as supplementary information on the web (Predicted_Amplicons_pathogen_sigs.xls). There was no single species-level signature for Dengue virus, so to detect the species requires a minimal set of 5 signatures, to detect any/all of the serotypes.

| Target  organism | NCBI Taxonomy node | #  Genomes available | Size of MSC soln | If not part of MSC soln=N | True  positives | Signature ID | Forward Primer | Probe | Reverse Primer | Strand | Amplicon length | Position relative to this target | Forward primer start position | Probe start position | Reverse primer start position |
| --- | --- | --- | --- | --- | --- | --- | --- | --- | --- | --- | --- | --- | --- | --- | --- |
|  |  |  |  |  |  |  |  |  |  |  |  |  |  |  |  |
| **Viruses** |  |  |  |  |  |  |  |  |  |  |  |  |  |  |  |
| Dengue 1 | [11053](http://www.ncbi.nlm.nih.gov/Taxonomy/Browser/wwwtax.cgi?id=11053) | 47 | 1 |  | 47 | group469_64 | GCCATAGTACGGAAAGAGCTATGC | CTGTGAGCCCCGTCCAAGGACG | CAGCTTCCATGGGTTGCA | + | 152 | gi|8927332 | 10340 | 10367 | 10474 |
| Dengue 2 | [11060](http://www.ncbi.nlm.nih.gov/Taxonomy/Browser/wwwtax.cgi?id=11060) | 57 | 2 |  | 28 | group57_661 | CACGTGTGACATGCCACATC | TTTGCTTGATTCTGTAGGCTCCATCTTCCA | CCCATGGGAAAGGCTGAA | - | 130 | gi|77024328 | 4665 | 4574 | 4555 |
|  |  |  |  |  | 39 | group287_278 | TAGGTCGCCTGATCACAGTCA | AGCAGAACCTCCATTCGGAGACAGCTACAT | TGTCCCGGGTCTACTCCTATGA | + | 115 | gi|77024340 | 1979 | 2040 | 2072 |
|  |  |  | OR alternate MSC soln | | | |  |  |  |  |  |  |  |  |  |
|  |  |  | 2 |  | 55 | group598_3392 | CTTCCAGGCCCCTTCTGA | CCACATGTGGAACGAGCGCCAC | CGTCCACATGGGTAACTTATGG | - | 143 | gi|51233484 | 775 | 715 | 650 |
|  |  |  |  |  | 56 | group1215_3979 | ATGATTGGTGCATTGCTCTGA | AGCTGCTTCACCCATCTCTACTCGAGTTGA | CCAGCAAGCATAGCAGCTAGAG | - | 128 | gi|9280544 | 5499 | 5422 | 5392 |
| Dengue 3 | [11069](http://www.ncbi.nlm.nih.gov/Taxonomy/Browser/wwwtax.cgi?id=11069) | 70 | 1 |  | 70 | group544_1654 | AGGCTGCAAACCGTGGAA | TGGTTAGAGGAGACCCCTCCCATGACA | AAGGAGGTACAGCTTCCCTCAGT | + | 117 | gi|9626683 | 10432 | 10476 | 10526 |
|  |  |  |  |  | 70 | group544_2584 | GACCTTAATAGCCCCCGACTTCT | CAATTTCATTGGTCCTTGGCCATTCAGC | GGCGAAGAGATTCTCAAGAGGAT | - | 154 | gi|82940933 | 312 | 205 | 181 |
| Dengue 4 | [11070](http://www.ncbi.nlm.nih.gov/Taxonomy/Browser/wwwtax.cgi?id=11070) | 11 | 1 |  | 11 | group30_2087 | GTGGACCGACGAGGACAGT | CAAATCGGAAGCTTGCTTAACACAGTTCTAACAGTT | GGGTTGATACGCGGTTTCTC | + | 160 | gi|53653744 | 15 | 36 | 155 |
| Human adenoA | [129875](http://www.ncbi.nlm.nih.gov/Taxonomy/Browser/wwwtax.cgi?id=129875) | 1 | 1 |  | 1 | group1_11 | TTCCGGAACCGCCTACAA | CACCAAAAGGCGCTCCTAATGCTTCA | GGCGCCTGAGCAAAGGTAT | + | 96 | gi|9626621 | 18087 | 18113 | 18164 |
|  |  |  |  |  | 1 | group1_17 | TTTCTTACATGGGCATCTTGCAT | CGAGACTGCATTTACCTTTTCCTGTTACATACCTG | CAAACACCCATCCCAGTTTG | + | 145 | gi|9626621 | 23806 | 23895 | 23931 |
|  |  |  |  |  | 1 | group1_25 | CCGGACCCCACATGATTT | CTGCTAGAACAAGCCGCTCTCACCG | GAGGGTTAAGTTGATTACGTGGTGTA | + | 108 | gi|9626621 | 25718 | 25774 | 25800 |
|  |  |  |  |  | 1 | group1_45 | CAGAGTATCCACACAGGATGCA | ACCAAGCCATGCTATGCAGGCAGC | TGTATTCCTCCTCCTCCTGTAAGTC | + | 87 | gi|9626621 | 32508 | 32538 | 32570 |
|  |  |  |  |  | 1 | group1_7 | GAGGCTGGTTTAATATACAACGCTTT | CGTGGCACGCTATAACAGTACCAATGTGC | GAGCCAGCGCCTCCTTTAC | + | 112 | gi|9626621 | 11861 | 11896 | 11954 |
| Human adenoB | [108098](http://www.ncbi.nlm.nih.gov/Taxonomy/Browser/wwwtax.cgi?id=108098) | 20 | 2 |  | 18 | group31_178 | GAAGAAGAACGATGGGAATCATAATC | TCGCTCCGTGCGACTGCTGTTT | TTAAGGGCTATTAAAATCATGCTTCAG | + | 155 | gi|31540430 | 32079 | 32162 | 32207 |
|  |  |  |  |  | 18 | group31_181 | TCAAAAACACACTACCCACATACATG | TCTCTTTTGGCATGTGCATATTAACAATCTGTCTG | GATTAACCAACGTTGTCCATGGT | + | 86 | gi|31540430 | 32428 | 32455 | 32491 |
|  |  |  |  |  | 18 | group31_369 | CAAGTGATTCATGGTCGAGAGAA | TCTTCATTGTCATTGTAATCAGCAGGGTTCACT | ATTTGTCTGTACCATGGACAACGT | - | 158 | gi|78059380 | 33160 | 33121 | 33025 |
|  |  |  |  |  | 12 | group38_266 | CCCACTATGTACAGTTCTATGAATCGA | TGCCCTTGACATGAGGCAGCTTCTTAA | CCTCGAACACTATGCTCTCTCTGA | + | 108 | gi|56160595 | 7425 | 7456 | 7509 |
| Human adenoC | [129951](http://www.ncbi.nlm.nih.gov/Taxonomy/Browser/wwwtax.cgi?id=129951) | 6 | 1 |  | 6 | group1_33 | AGTAGTATAGCCCCACCACCACAT | CAAACTCACAGAACCCTAGTATTCAACCTGCCA | TGTTACCCATGATATGATGCTTTTTAA | + | 159 | gi|9626158 | 32858 | 32909 | 32990 |
|  |  |  |  |  | 6 | group1_40 | CCCTGTAGCCGGAGGGTTA | TCATGCAAGACCCCGCTTGCA | GTCCCTGTTTCCGGAGGAAT | + | 142 | gi|9626158 | 10874 | 10974 | 10996 |
| Human adenoD | [130310](http://www.ncbi.nlm.nih.gov/Taxonomy/Browser/wwwtax.cgi?id=130310) | 6 | 1 |  | 6 | group10_116 | ACGCATGCAAGGTGTGGTT | TCCTATATAAGTGGCAACACCTGGGCACTG | CTACAAGCCGGCGTGTCTT | + | 150 | gi|134141818 | 1476 | 1518 | 1606 |
|  |  |  |  |  | 6 | group10_160 | ACCAGCCCATTTCACAGTGTAA | CACAGTTTCCTGGCGAGCCAAACG | CAGAGGACGGCTTCATCTCAA | + | 158 | gi|88810171 | 32233 | 32335 | 32370 |
|  |  |  |  |  | 6 | group10_225 | GCCCAGGTGTTGCCACTTA | AGGACTAAGCCCCGCCCATGAGTCAT | ACGCATGCAAGGTGTGGTT | - | 68 | gi|88810171 | 1520 | 1491 | 1471 |
|  |  |  |  |  | 6 | group10_31 | AGCTGCGGACTCATCTACTTTGA | CCCAACGGTCCTGCACACGGA | AGACTCGGTGGTGCCCTCTAC | + | 93 | gi|56237058 | 26265 | 26304 | 26337 |
| Human adenoE | [130308](http://www.ncbi.nlm.nih.gov/Taxonomy/Browser/wwwtax.cgi?id=130308) | 12 | 1 |  | 12 | group6_10 | GGCACAGGGAACTCTTGCA | CCTCGCACGGAACTTACATTGTGCATG | ATCCGGTGCTGCCTGATT | + | 106 | gi|51527264 | 33931 | 33979 | 34019 |
|  |  |  | OR alternate MSC soln | | | |  |  |  |  |  |  |  |  |  |
|  |  |  | 2 |  | 9 | group7_1 | CAAGCCCTGGCCTGAGTT | CAATGTCATGACCAGGTGCAATATGCATC | AGGTTGCACTGGTAGGGCATAA | + | 96 | gi|51527264 | 3002 | 3026 | 3076 |
|  |  |  |  |  | 3 | group9_104 | TAGAGGCCCACCGAAGCA | CAGGTAAGAGACCCCCTCCGATGAGTTCT | CTTTCACCAGACTAGACAGCTGCTA | - | 82 | gi|41764064 | 1690 | 1652 | 1626 |
|  |  |  |  |  | 3 | group9_58 | TTCAGAGCAGGACTCATGGAGAT | TTGGAAGACTTTCACCAGACTAGACAGCTGCT | CTTATAATCCTGCTTGGCCCTATAGAC | + | 159 | gi|41763965 | 1584 | 1617 | 1716 |
| Human adenoF | [130309](http://www.ncbi.nlm.nih.gov/Taxonomy/Browser/wwwtax.cgi?id=130309) | 2 | 1 |  | 2 | group3_113 | TCCAACATGCAACAAACGTACAC | CCCTACCCCAGGAATCCACGCA | ACCACTCAATTAACCTTGCATGGT | - | 109 | gi|9626553 | 2519 | 2484 | 2433 |
|  |  |  |  |  | 2 | group3_34 | GCTTTTCCCATTTAGACAATATCATGT | CCTCACCTCGCTCACATAATGCATATTCAAA | GGACTCCTGGACTTTTAGAGTGTGA | + | 141 | gi|9626553 | 32502 | 32584 | 32618 |
|  |  |  |  |  | 2 | group3_62 | CATTTATTACACCCCCGTTTGC | CGGCTTGCAAGAAAAACCACCGG | CCGTGCCCAGTTTTAAGGTT | + | 126 | gi|83657216 | 30013 | 30044 | 30119 |
| Canine distemper virus | [11232](http://www.ncbi.nlm.nih.gov/Taxonomy/Browser/wwwtax.cgi?id=11232) | 11 | 1 |  | 11 | group1_1 | TCCCTCATGATGCTATATCAACAGAT | TGTTCAAAACAAATTTAGTGCAGGGTCCTACCC | CCCATAGCATAACTCCAGAGCAA | + | 122 | gi|9630645 | 999 | 1064 | 1098 |
|  |  |  |  |  | 11 | group1_225 | GGGCATGATCCGCTCATC | CAAGACTCATGTTCCGTCCAGTTAGCGAGA | AGCCCACATGTGGTTCCTTAA | + | 75 | gi|5733642 | 12393 | 12414 | 12447 |
|  |  |  |  |  | 11 | group1_141 | TTTGTGTCAACAGAGGCCTACCT | TCGGTATCCGAACTTCACCCGGG | TTGATGAAAGGATGTGCCCTACTA | + | 139 | gi|37654450 | 14877 | 14968 | 14992 |
|  |  |  |  |  | 11 | group1_186 | GGCGAACTGCAGGTGTCA | CATCTTAACTCCGTGGAAGAAGGTCCTTACGA | GACTTGATTTGCACTGAACACACTT | + | 116 | gi|5733642 | 3736 | 3791 | 3827 |
|  |  |  |  |  | 11 | group1_211 | CTACCCCATGTGCAACCAAA | CCATCTTATGGGCGGTTGACATTACCTCTAGA | CATTCAGTATAACCGGACCGTATG | + | 143 | gi|49182263 | 8236 | 8288 | 8355 |
| Coxsackie B4 | [12073](http://www.ncbi.nlm.nih.gov/Taxonomy/Browser/wwwtax.cgi?id=12073) | 3 | 2 |  | 2 | group1_12 | CTCTCTCCACGGACTCTTCTGTT | TGCGTGTCCCTCAACATGCGTACAG | AACTAATGTCATAGTCCCAGCTGAAG | - | 158 | gi|94449174 | 2453 | 2395 | 2318 |
|  |  |  |  |  | 2 | group1_7 | GCACTACAGGTATGTTGTTGATGACA | CCCAGCTGAAGCTCAGAAATCGTGCT | GTCCCTCAACATGCGTACAGA | + | 160 | gi|94449174 | 2255 | 2333 | 2394 |
|  |  |  |  |  | 1 | group2_5 | AATCTGTGGAGCGAGCAATG | AGATCCCTGCCCTAACGGCTGTGG | TCTACTTGAGAAGTGTGCCCAGTT | + | 115 | gi|17224874 | 2462 | 2528 | 2553 |
|  |  |  |  |  | 1 | group2_1 | GGTATACGATCCACGTACAATGCA | CGCGGAAAATGCGCCCACA | CCCTCCGCATAGATCACCAT | + | 134 | gi|17224874 | 1265 | 1359 | 1379 |
| Cytomegalovirus | [10358](http://www.ncbi.nlm.nih.gov/Taxonomy/Browser/wwwtax.cgi?id=10358) | 7 | 1 |  | 7 | group14_2521 | TGTTTACGGATATACCGGAACGA | CGGATTTTACCTACTGGTCCCGTACTTCGG | CCCACCCGCTTGACTATAACC | - | 88 | gi|20026600 | 84402 | 84359 | 84337 |
| HumanHerpes1 | [10298](http://www.ncbi.nlm.nih.gov/Taxonomy/Browser/wwwtax.cgi?id=10298) | 1 | 1 |  | 1 | group1_11 | GGAGACCCCAAAACGACAGA | CCTCCCGACGGATGCGCG | CAGATGGGACGCCATACACA | + | 152 | gi|9629378 | 17977 | 18060 | 18109 |
|  |  |  |  |  | 1 | group1_12 | TGTGTATGGCGTCCCATCTG | CGGACAGACTCAAGCACACACGGG | TGACGACAAGATGTCGTTTCTG | + | 146 | gi|9629378 | 18109 | 18159 | 18233 |
|  |  |  |  |  | 1 | group1_14 | CTCACCGGCGGCTAAGTC | TCCACCCTGCCCATTTCGTACGA | AACCGACAGATGTACTCGCTGTA | + | 117 | gi|9629378 | 97525 | 97595 | 97619 |
|  |  |  |  |  | 1 | group1_1 | AACACATACACATGGCCCCTTT | CCCACAACAAACACACAAGGACCGG | GGGCCTGCGTATGAGTCAGT | + | 155 | gi|9629378 | 2630 | 2713 | 2765 |
|  |  |  |  |  | 1 | group1_8 | CGGGTAAGTAACAGAGTCTGACTAAGG | CCCCTGTCGTTTGGGTCCCCC | GACACCTGCTTCTCCCCAATA | + | 160 | gi|9629378 | 6740 | 6853 | 6879 |
| HumanHerpes2 | [10310](http://www.ncbi.nlm.nih.gov/Taxonomy/Browser/wwwtax.cgi?id=10310) | 1 | 1 |  | 1 | group1_12 | AGCTGACGGCCAGTCACA | CCTTATATGTGCACGGCAAATACTTCTACTGCAAC | CGGAACCACTTGGGTTGAC | + | 113 | gi|9629267 | 116054 | 116081 | 116148 |
|  |  |  |  |  | 1 | group1_13 | CTCTCTTGCCGGGTTTTAGTCT | CCCGGCGCCTATCCACTCCC | GTAAATGCCGCCCCTTTG | + | 160 | gi|9629267 | 124989 | 125110 | 125131 |
|  |  |  |  |  | 1 | group1_16 | CTGAAAACGACGGACATGTTTCT | CGCGGTGCACACACCGGA | GCGCAGACATCGCCATAC | - | 68 | gi|9629267 | 148001 | 147975 | 147956 |
|  |  |  |  |  | 1 | group1_1 | GTAAATGCCGCCCCTTTG | CGCCGGGTATAAGGCAGCCCC | CTCTCTTGCCGGGTTTTAGTCT | + | 160 | gi|9629267 | 2102 | 2134 | 2240 |
| HumanHerpes3 | [10335](http://www.ncbi.nlm.nih.gov/Taxonomy/Browser/wwwtax.cgi?id=10335) | 19 | 1 |  | 19 | group3_100 | AAACGTACGCTCCTACTACCGAGTAT | AAAACGGTATGAAACGCTATTACCCGCCTT | GCCTCCCGGTGGTAATCC | + | 110 | gi|9625875 | 32056 | 32117 | 32148 |
|  |  |  |  |  | 19 | group3_1010 | CTCGAATTTTTGTTGCGGTACATAG | CCTTTAAATCTCGCGCCGCCTGA | CACTCACGCATTTACCTGGTGTAC | - | 158 | gi|9625875 | 92320 | 92280 | 92187 |
|  |  |  |  |  | 19 | group3_1084 | CAATGAGCGCGGACAGTTT | TCCATATGCAGTAAACGTAACCCCCAAGAAA | CTCAGCCGTATCTCCCGATATT | - | 116 | gi|9625875 | 41097 | 41023 | 41000 |
|  |  |  |  |  | 19 | group3_1022 | GTGTTGGGAGACATGGGAACT | CCACGACACCCGAGGTGGAAGC | CAAAGTGAACATCATCACCTGAATTA | - | 153 | gi|9625875 | 87874 | 87809 | 87742 |
|  |  |  |  |  | 19 | group3_1036 | AACTACATGGAATTTCAGAGTCAAAGG | CGTAACATATTTTAACGCATCCCCGTGACC | GTGGATTATTTGGCACTCATTTGT | - | 157 | gi|9625875 | 52325 | 52291 | 52195 |
|  |  |  |  |  | 19 | group3_1080 | ACTCGCCACAACTCACAATTTAGA | CGGCACCTCCGACGCTTCG | CTCATGCATATGGTGCAGGTATT | - | 157 | gi|9625875 | 104548 | 104441 | 104415 |
| HumanHerpes4 Epstein-barr | [10376](http://www.ncbi.nlm.nih.gov/Taxonomy/Browser/wwwtax.cgi?id=10376) | 4 | 1 |  | 4 | group1_101 | GGGCTTTGGGTTCCATTGT | CTTTCCTGGCCAACGTGAGGGTCC | GGTTCAACTCCAGGGTCTAGCA | + | 134 | gi|139424470 | 148541 | 148565 | 148653 |
|  |  |  |  |  | 4 | group1_116 | CGAACGATGAGGAACGTGAA | CACCGCCTTATGAGGACCCATATTGGG | GAGTGACGGTCGCCATTG | + | 82 | gi|139424470 | 167720 | 167756 | 167784 |
|  |  |  |  |  | 4 | group1_266 | TGCAATGCCCACCCACTA | CCCGGCGGACCCGATCAG | GCCAAGATAATGGATTGAGTAAGCAT | - | 80 | gi|139424470 | 109120 | 109090 | 109058 |
|  |  |  |  |  | 4 | group1_325 | GTGGGCGCTCCTCACTTT | CCAAACCCATGTAAGTCATGTGTCAGAACG | ACCAACAGGTGTTGCCTTGTTA | - | 148 | gi|139424470 | 1637 | 1535 | 1507 |
|  |  |  |  |  | 4 | group1_425 | CAAGTACAGAGCCCACCACAGAT | ACTGCGGCCCCGCTGGAC | GTAACCGGTGGGCTCGTAAA | - | 136 | gi|139424470 | 77594 | 77502 | 77481 |
| HumanHerpes6 | [10368](http://www.ncbi.nlm.nih.gov/Taxonomy/Browser/wwwtax.cgi?id=10368) | 3 | 1 |  | 3 | group3_1017 | ATACTCCGCCAGACTGGAACA | CTACAGACGCCGGAGAAATCCAAGACTTG | TTCGTTACGGTAGCATATGGTGACT | + | 159 | gi|9628290 | 55362 | 55465 | 55496 |
|  |  |  |  |  | 3 | group3_10508 | AGCGTATAGTTATCGAGGGACACAT | CTGCGTTGCATAAAAACTTGGACTGCTAAAAATTAC | ATTCAGGCTAGCATGGTGTGAA | - | 137 | gi|9628290 | 119188 | 119143 | 119074 |
|  |  |  |  |  | 3 | group3_10819 | GCCGAATGTTTCCAGAAAAGA | TTACAATCGACCATCAAAATATAAAGAGCACAGCAC | TGTATTGTAGCAGCTGGCGAAA | - | 104 | gi|9628290 | 101700 | 101640 | 101617 |
|  |  |  |  |  | 3 | group3_12257 | GGTGCATAAACGGGACTATGG | TCAAATAAGTCCCGAGAAAGACGTCAGAGCTATC | ACGTCCAACGACCCCAAT | - | 133 | gi|9628290 | 13408 | 13322 | 13296 |
| HumanHerpes7 | [10372](http://www.ncbi.nlm.nih.gov/Taxonomy/Browser/wwwtax.cgi?id=10372) | 1 | 1 |  | 1 | group1_10 | ATACTGACGGGCCGCTCAT | CACGTCCGTTAAAACAACGCCACATG | ACCGCAGACTGCGTCCAT | + | 93 | gi|51874225 | 5369 | 5416 | 5444 |
|  |  |  |  |  | 1 | group1_105 | GTGAGACCGACATTAATGACACTGA | TGTCCTCTAGCTAATGCTTTGCTGCAACAAA | CCACTTCCGCAAAGTAAGCA | - | 157 | gi|51874225 | 67015 | 66938 | 66883 |
|  |  |  |  |  | 1 | group1_109 | AAGAGGTAAACACAGCTAACGAGACA | AACTCGCCACTGAAAAACTTGGAACACACTT | ACCGGGAAACCCGAAGTT | - | 145 | gi|51874225 | 30309 | 30209 | 30190 |
|  |  |  |  |  | 1 | group1_24 | ACAGCTGTAAGCTGCAGGAAAGA | CACCTCTAAGCATAACCTGTCGGGCACA | TTTACGGCTGCGTGTCTCA | + | 136 | gi|51874225 | 8802 | 8890 | 8919 |
| Japanese encephalitis | [11072](http://www.ncbi.nlm.nih.gov/Taxonomy/Browser/wwwtax.cgi?id=11072) | 43 | 2 |  | 37 | group42_324 | CCCAGGCGGCAAAGTTTAC | CCCTCAAACTTGGTGACTACGGAGAAGTCA | AGTCCACTCCTTGGCTCACAGT | + | 103 | gi|9626460 | 1462 | 1507 | 1543 |
|  |  |  |  |  | 37 | group42_461 | GAGAGCGAGGTCATGAAACCA | CACTCCTTGGCTCACAGTCCAGTGTGA | TCAAACTTGGTGACTACGGAGAA | - | 137 | gi|45934772 | 1626 | 1534 | 1510 |
|  |  |  |  |  | 37 | group42_666 | ACACTCGTCAGTGCTTTCCTCTCTA | CAGCCACACACGGGTTGATGTGATACC | ATTTGTTGTAGATGGACCTGAGACA | - | 142 | gi|46519720 | 2990 | 2958 | 2873 |
|  |  |  |  |  | 38 | group248_605 | GCAGCTGTTCCAAGTGTTGTG | CACCTGCCCACTCCCCCTTTGG | TGATGGACGTGATATCACGAGAA | - | 147 | gi|81687247 | 9578 | 9477 | 9452 |
| Mumps virus | [11161](http://www.ncbi.nlm.nih.gov/Taxonomy/Browser/wwwtax.cgi?id=11161) | 17 | 1 |  | 17 | group119_253 | GACGTCCCATCGAGGGATT | AACACCTGATCCCATTGAATTGGTACATGG | GCTCACATTCATCTGACCCAATTA | + | 84 | gi|55775563 | 11919 | 11941 | 11979 |
|  |  |  |  | N | 16 | 243 | GATTAACGGTTATAGGAGGAGCCATAA | TGGCAATGCCCCCAAAAGCCT | TCTGAATTATCATGCCGCATTT | + | 102 | gi|55775563 | 9661 | 9715 | 9741 |
|  |  |  |  | N | 16 | 170 | ACAGTTTGTTCTGCCTCACTCAAG | CGCCCATTGCTTGCTGCACG | CAAAACTTCCATCTGAACTGCTCTTAC | + | 93 | gi|55775563 | 3795 | 3831 | 3861 |
| Newcastle diseasevirus | [11176](http://www.ncbi.nlm.nih.gov/Taxonomy/Browser/wwwtax.cgi?id=11176) | 38 | 4 |  | 32^1^ | group174_256 | TCTTTTCTACTCTGCGTTCCATCA | TGACACCCAAAATCGGAAGTCTTGCA | ACAACCTAAGGGAGTTGCACTCA | + | 83 | gi|45511239 | 7076 | 7107 | 7136 |
|  |  |  |  |  | 13^1^ | group474_488 | ATATCTCGGGTCGAAGACTCAAGA | CATATGTCACCACATGTGAAAGCAGCACTAAGG | AAGCCCAGATTAACACGGATGA | + | 113 | gi|45511204 | 11959 | 12014 | 12050 |
|  |  |  |  |  | 14 | group559_542 | CTCACTGAGTACCTACTGTCAGATGCT | CCAGCCAACCTATATTACATGTCTAGGAAGAGCCTT | CTCTGTCCTCTCGTTCCCTGAT | + | 154 | gi|45511239 | 13238 | 13328 | 13370 |
|  |  |  |  |  | 1 | group124_1000 | ATCACACACTGCGAGGAGTGTT | CAGTTTCTGCAGTATTTGATTCCATTGCACG | TGCTGCTAGAGCTTACCCTAGTTACTC | + | 123 | gi|73476661 | 7865 | 7925 | 7961 |
| Sendai virus | [11191](http://www.ncbi.nlm.nih.gov/Taxonomy/Browser/wwwtax.cgi?id=11191) | 13 | 1 |  | 13 | group26_337 | CTTCCCATTAATGGACGTGAATC | TGATTTCCGCTACTACCCTAATGTTGTGGCA | AGCTTCCTGATCCTTCCTATGTTCT | + | 159 | gi|151935426 | 4553 | 4655 | 4687 |
|  |  |  |  | N | 12 | 601 | GAAAGGATCAGGCCGCATA | CAGACTTTAGGGCACGTCTTGCAAACACA | CATCCCGAGACGCGAGTT | - | 94 | gi|56378309 | 2955 | 2899 | 2880 |
| St. Louis Encephalitis | [11080](http://www.ncbi.nlm.nih.gov/Taxonomy/Browser/wwwtax.cgi?id=11080) | 2 | 1 |  | 2 | group17_83 | TCGCTGACTCTACTGGCTGTTG | CCAGCGTGCAAGCCGATTCG | AGATGCCTCCTCCACATTTCA | + | 118 | gi|123205971 | 2406 | 2452 | 2503 |
|  |  |  |  |  | 2 | group17_172 | TCATGATTCGGTAGATTCCTGGTT | TGACGGCACGTCCCACAATGC | CTGGGCTTGAGTGCACGAT | - | 160 | gi|123205971 | 4651 | 4602 | 4515 |
|  |  |  |  |  | 2 | group17_233 | GTTTCGTAGGACTGCTCCTTCAGT | CATGATTCGATAGATTCCTGGTTTTGTCTCACA | CCGTCACCAAAAGTGTATCCAA | - | 159 | gi|123205971 | 4752 | 4641 | 4617 |
|  |  |  |  |  | 2 | group17_39 | GGGTTGAAGAGGATACTTGGAAGT | CGTGCGGTTCATACTAGCCATCCTGAC | CTGTAGAGCTGTGAATCGGAAGAA | + | 96 | gi|123205971 | 183 | 227 | 255 |
| Papillomaviridae | [151340](http://www.ncbi.nlm.nih.gov/Taxonomy/Browser/wwwtax.cgi?id=151340) | 107 | 72 |  |  |  |  |  |  |  |  |  |  |  |  |
| Human papillomavirus type 16 | [333760](http://www.ncbi.nlm.nih.gov/Taxonomy/Browser/wwwtax.cgi?id=333760) | 14 | 1 |  | 14 | 1879 | CCGTACCCTCTTCCCCATT | CCATTAACAGGTCTTCCAAAGTACGAATGTCTACGT | CTTCGGTTGTGCGTACAAAGC | - | 151 | gi|9627100 | 886 | 779 | 754 |
|  |  |  |  |  | 14 | 1883 | ACACAATTCCTAGTGTGCCCATTAA | CTACGTGTGTGCTTTGTACGCACAACCG | AACCGGACAGAGCCCATTAC | - | 135 | gi|9627100 | 808 | 757 | 698 |
|  |  |  |  |  | 14 | 1886 | TCCACTACAGCCTCTACATAGAACCA | CCATTACATCCCGTACCCTCTTCCCCAT | GAAGACCTGTTAATGGGCACACTA | - | 143 | gi|56463005 | 916 | 887 | 799 |
|  |  |  |  |  | 14 | 1892 | GACAATCACCTGGAGTTACTGCAA | TCCTTTGCCCCAGTGTTCCCCTATAGG | CAATTGTGTTTAATTGGTTGCAAAC | - | 94 | gi|56463005 | 6172 | 6129 | 6102 |
| Human papillomavirus type 71 | [120686](http://www.ncbi.nlm.nih.gov/Taxonomy/Browser/wwwtax.cgi?id=120686) | 5 | 1 |  | 5 | 1981 | TTGGACAGAAACTACACTGCTGTTG | CCCCAAACAATGGGTATTGCACGGT | GTGTGCCTGGCAGTTATTTGG | - | 160 | gi|12084981 | 7925 | 7858 | 7790 |
|  |  |  |  |  | 5 | 1977 | TTGGACAGAAACTACACTGCTGTTG | CGAACATAGTCAGCACAACTATATGCCCCAA | TTACTCACCGTGCAATACCCATT | - | 98 | gi|12084981 | 7925 | 7877 | 7852 |
|  |  |  |  |  | 5 | 1982 | GTGTAGGCCTGCTGGACACA | CCCGCCCTAGTGGCTGCAGTTCTAA | CCCTATACAACCCACCGAACA | - | 151 | gi|12084981 | 5542 | 5433 | 5411 |
| Human parainfluenza virus 1 | [12730](http://www.ncbi.nlm.nih.gov/Taxonomy/Browser/wwwtax.cgi?id=12730) | 1 | 1 |  | 1 | group1_4 | AACAGACAGGAATTGGCTCAGA | TCCTCTGGACCCACACGATTTCTGG | GGTTCCCCTACGGGACATC | + | 110 | gi|19718363 | 7290 | 7355 | 7381 |
|  |  |  |  |  | 1 | group1_13 | GTCTTTGTCGACTGGGAGACATT | CCTTGTCTCAGTCGGCTGGACCATG | AGATTCGATCCATGCTCCTCTACTA | - | 112 | gi|19718363 | 4138 | 4075 | 4049 |
|  |  |  |  |  | 1 | group1_16 | TCGACTCCAAATGTTATGTCTGTATG | CGCACCATCCCCTCCTGAAAGG | CAAAAGAGAGACTAAGACACCATCTGA | - | 124 | gi|19718363 | 1386 | 1319 | 1288 |
| Human parainfluenza virus 2 | [11212](http://www.ncbi.nlm.nih.gov/Taxonomy/Browser/wwwtax.cgi?id=11212) | 5 | 1 |  | 5 | group5_11 | CAGGACTTGGGATTATTGAGACCTA | CCATTTGCACACTAGCTCATCTTGTTGTGTTAGA | TCTGAGGAACCTCTCCATTGC | + | 150 | gi|19525721 | 12832 | 12899 | 12961 |
|  |  |  |  |  | 5 | group5_16 | CCATTACATACTCACCACGAAATCTG | CCAGACCTATCCTTAAGTTCAGATCACGTTTTGTG | GGGATTCAGATTTGCATAGCAAT | + | 126 | gi|26655522 | 4003 | 4042 | 4106 |
|  |  |  |  |  | 5 | group5_41 | TACATCCCAACTTTTGTCTCCATTAC | CACGAAATCTGAATTACCAAGTTGCCAGACCTAT | ACACAAAGCGTGATCTGAACTTAAG | + | 94 | gi|26655521 | 3984 | 4018 | 4053 |
|  |  |  |  |  | 5 | group5_55 | TCTGAGGAACCTCTCCATTGCT | AGGCAACCATCTACACTTCTAACACAACAAGATGAG | CAGGACTTGGGATTATTGAGACCTA | - | 150 | gi|19525721 | 12960 | 12914 | 12832 |
|  |  |  |  |  | 5 | group5_67 | GGGCACACCTGGGATTACA | CACTCGAAGACTCTGACCTGATCTCCGAC | AGCCAGTCTCCTCAGCTGGAT | - | 156 | gi|67906100 | 2573 | 2543 | 2436 |
| Human parainfluenza virus 3 | [11216](http://www.ncbi.nlm.nih.gov/Taxonomy/Browser/wwwtax.cgi?id=11216) | 2 | 1 |  | 2 | group2_10 | TAGCTGCTGAATGCTTCTATACATTCT | TCCACCTAGAAATGCCCCTTTCGTCA | ATATCCCTCTTCCCAGCCATATC | - | 88 | gi|1853985 | 6058 | 6021 | 5997 |
|  |  |  |  |  | 2 | group2_6 | CCCTCCAAGTCAGACTCCCTTTA | TTCCCAGCCATATCATGACGAAAGGG | TTGACGTCTGCTCCACCTAGAA | + | 157 | gi|1853985 | 5901 | 6006 | 6036 |
|  |  |  |  |  | 2 | group2_15 | GTCTCTTCAAGCTTTCTTTAGCTTCGT | CAGCATCACGTGCTACTGCTTGTCCTAGCT | AATAGAGCCATGCAACAGTATGTGA | - | 160 | gi|10937870 | 1270 | 1195 | 1137 |
| JC polyomavirus | [10632](http://www.ncbi.nlm.nih.gov/Taxonomy/Browser/wwwtax.cgi?id=10632) | 380 | 2 |  | 296 | group33_79 | CCACCCCCTGTAATTCTAAAGC | TGGCTTCCCTGCACCATTGTCATG | TGTGCACTCTAATGGTCAAGCA | - | 113 | gi|55953243 | 1910 | 1844 | 1819 |
|  |  |  |  |  | 369 | group36_106 | AACTTTATGATCCCAGTCAGCAAAA | AGCTACAGCCCCCGGAGCTCCAGTA | GGCTATAGCTGCAATAGGCCTTACT | - | 160 | gi|9796432 | 932 | 842 | 797 |
|  |  |  |  | N | 376 | 57 | TATGAGTGGCCAAAGGAAATATAGGT | TCCCTTTGGGCAACCTGCCTTACC | GATTGTCTCCAGTTAGGCCCTCTA | - | 79 | gi|9796432 | 1344 | 1316 | 1291 |
|  |  |  |  | N | 379 | 18 | AGAAACTACTTGGGCAATAGTCAATTC | CCCTCTATGGTAAGGCAGGTTGCCCAA | GTGTATGAGTGGCCAAAGGAAAT | + | 150 | gi|32344889 | 828 | 913 | 955 |
| BK polyomavirus | [10629](http://www.ncbi.nlm.nih.gov/Taxonomy/Browser/wwwtax.cgi?id=10629) | 120 | 1 |  | 120 | group5_102 | CAATACCTTGATCCTAGGCATTGG | TTGCTACTATTTCCCAGGCTTTGTGGCA | AAATCTTTCTGTTCTTCTTTGCAATTC | + | 129 | gi|48869545 | 1038 | 1075 | 1140 |
|  |  |  |  |  | 120 | group5_1089 | GATTTTCAGTGGCTGAAATTGCT | CCTTGCTACTGTAGAGGGCATAACAAGTACCTCAGA | GAGCACCAGCAATTACAGCATATG | + | 156 | gi|9627180 | 691 | 755 | 823 |
|  |  |  |  |  | 120 | group5_1275 | AGATGGCCCCAACCAAAAG | CCCGTGCAAGTGCCAAAACTACTAATAAAAGGAG | CTCTGTTATAGCATCTACCCCAGTTTT | + | 143 | gi|77799529 | 1442 | 1504 | 1558 |
| WU polyomavirus | [440266](http://www.ncbi.nlm.nih.gov/Taxonomy/Browser/wwwtax.cgi?id=440266) | 6 | 1 |  | 6 | group2_4 | ATGTTGGTGTACCCACAACCAGTA | CATTTGCCTGCAACTGTAACTTTACAAGCCA | TATGTTAATATGGTGCAGGCCTGTTA | + | 290 | gi|148724565 | 2688 | 2720 | 2952 |
|  |  |  |  |  | 6 | group2_6 | GCAAGGGCCCTGTTTCTTC | CATAGTAACCTCTGTAACCTGCAGTTGCCCACT | GGTGTTTAATAAGCCAGCTGATGAC | - | 83 | gi|148724565 | 2651 | 2615 | 2587 |
|  |  |  |  |  | 6 | group2_3 | CTTGTAAAGTTACAGTTGCAGGCAAA | CATAGTAACCTCTGTAACCTGCAGTTGCCCACT | TGTTTAATAAGCCAGCTGATGACAT | - | 159 | gi|148724565 | 2722 | 2615 | 2589 |
| West Nile virus | [11082](http://www.ncbi.nlm.nih.gov/Taxonomy/Browser/wwwtax.cgi?id=11082) | 84 | 6 |  | 74 | group29_212 | CAAAGTCAGCAGTCTACGTCAGGTA | CCACTCAAGACGCAGTCGGAGGTCA | CCATCCAAGCCCCCTTCT | + | 129 | gi|9930133 | 674 | 720 | 785 |
|  |  |  |  |  | 74 | group29_1218 | GCTAGGCCTTGTGGCGTTT | CGGTCCATCCAAGCCTCCACATCAT | GATGTGGAAGTGGAGTGTTCATACA | - | 88 | gi|9930137 | 2579 | 2537 | 2510 |
|  |  |  |  |  | 74 | group29_465 | GCTCTGTTTGGAACGCAACA | CGGACTCTGCCACATCATGCGTG | TCCCAAGGTGCGTCCTTT | + | 149 | gi|9930133 | 7559 | 7587 | 7690 |
|  |  |  |  |  | 74 | group29_916 | GGTTGGGATCACATGCAATC | AGGCTAGAGCCAAGCATAACAGACTTGCTCCT | GCAGCTTGATCAGGGAGAAGA | - | 135 | gi|89148117 | 4191 | 4119 | 4076 |
|  |  |  |  |  | 6 | group72_200 | GCCTACACACCTTGGGCAAT | CACCATCACCCAAGGAGTACAAGAAGGGT | TCTGTAAACGCCAGTGGTGGTA | + | 141 | gi|11528013 | 4531 | 4619 | 4650 |
|  |  |  |  |  | 6 | group72_205 | ACACTCCAAAGGCGATATGGA | CCTTGTAAGAAACCCCCTTTCACGCAACT | AGTGGACGATGTTGCCTGAAG | + | 106 | gi|84028434 | 8272 | 8298 | 8357 |
|  |  |  |  |  | 1 | group101_1107 | ATCCGTTTGGCATGTTAATGTTG | TGATTCTAGCCTCCGTCCAGTGAGCG | ATGAGTACTGCTACGGAGGACACA | - | 102 | gi|30349725 | 6088 | 6055 | 6009 |
|  |  |  |  |  | 1 | group117_485 | GAGCCTTTAGATCGCTCTTTGG | AACGCCCGTGACAGGTCAATTGCTA | TTCCTCCCACAGCAAGGAA | + | 129 | gi|114204695 | 2308 | 2388 | 2418 |
|  |  |  |  |  | 1 | group170_846 | GGCACAAACCAGACCGTCTT | CCAGAGTTCCACGCTCTGTCGGGTATC | CTCCAGGTACCCATGATCCATT | - | 136 | gi|83699610 | 5683 | 5628 | 5567 |
|  |  |  |  |  | 1 | group5_431 | GGAGTGATCGAGAGTGAGCTCAT | CCAGTTACTTTGGCCGGTCCAAAGAGC | GCCTGGCCTCTTGTTGTGAT | + | 75 | gi|58702120 | 3172 | 3199 | 3227 |
| Yellow fever virus | [11089](http://www.ncbi.nlm.nih.gov/Taxonomy/Browser/wwwtax.cgi?id=11089) | 15 | 3 |  | 12 | group20_190 | GCTCACAGACCTCTGGAGGAA | CACTCCGGTCTTTCCCTGGCGTC | AGGGAACAAATAGTGGGACCATAT | - | 80 | gi|9627244 | 10791 | 10757 | 10732 |
|  |  |  |  |  | 2 | group11_115 | GAAAGGTTACACTTTGGGCAGAGA | ATTGTGACCTTCAAGGATAAGACTGACATCCACA | TTCACACTTGGCTGGCTCTAGT | + | 124 | gi|63147728 | 7940 | 8007 | 8042 |
|  |  |  |  |  | 1 | group47_451 | GGCTCCAGTCGGTGAACAT | TCCATCCCAAGCTTTGGACATTCATAGG | TGGCAGAGATGGTCATGAGAA | - | 96 | gi|63147726 | 8033 | 7978 | 7956 |
| Rabies virus | [11292](http://www.ncbi.nlm.nih.gov/Taxonomy/Browser/wwwtax.cgi?id=11292) | 26 | 2 |  | 24 | group65_1141 | TTCAGGGAGACTGTCCACCTCTA | CGCACTTGGATTGACAAAGATCTTGCTCA | AAAAACTAACACCCCTCCTTTCG | - | 189 | gi|51860793 | 1644 | 1515 | 1478 |
|  |  |  |  |  | 22 | group96_1039 | AAGTTTCTCTCAACCCTCTGGAGTAG | CCTCTCGCTCATAGCGTAGCACGTGTTG | CAACCACTATGAGAGAAGGCAACA | - | 199 | gi|113883018 | 10060 | 9931 | 9887 |
|  |  |  |  |  |  |  |  |  |  |  |  |  |  |  |  |
|  |  |  |  | N | 22 | 822 | CCGGAAACTCGAAGATCTGAGA | CCATGTCTGTGCATAAAGCATCAATGGC | ATTTGACCCAAGACGGGAAGA | - | 198 | gi|9627197 | 9411 | 9275 | 9235 |
|  |  |  |  | N | 24 | 674 | CAACTCAGAGATCCACGGGATTA | CAGTTCCTCACCCTTCTGAGATGTTGGG | TTGCTCCACAAGTGCAAGAAAT | + | 200 | gi|9627197 | 8627 | 8758 | 8805 |
|  |  |  |  | N | 21 | 685 | AACATATGTTGAAGTGCCTCAGGAT | AGCCACTTTAGACAGAGTTCCCCTCTGTAACTCA | AGAATCTTTTCTAGTCCACAAGATGGT | - | 191 | gi|9627197 | 11412 | 11282 | 11246 |
| Human respira-tory syncytial virus | [11250](http://www.ncbi.nlm.nih.gov/Taxonomy/Browser/wwwtax.cgi?id=11250) | 9 | 2 |  | 7 | group1_1226 | GAAGAAATAAATGATCAGACGAACGA | CACATTAGTAGTAGCAAGTGCAGGACCTACATCTGC | CTTAAACCAACCATGGCATCTCTT | + | 161 | gi|60549163 | 2706 | 2795 | 2843 |
|  |  |  |  |  | 7 | group1_1434 | CATGTAAATGCTGGTAAATCAACCA | TGTAAGGCCAGAAGCACACCAGTCACAC | TTTATACCACTCAGTTGATCCTTGCTT | + | 161 | gi|60549163 | 7202 | 7307 | 7336 |
|  |  |  |  |  | 7 | group1_3268 | ACTTAGTCCTTACAATAGGTCCTGCAA | CCAAAAATTTCATCATGCCTAAGAAGGCTGATAAA | GGGTAACAAAGAAAGGGTATCAAACTT | + | 166 | gi|1912287 | 14483 | 14564 | 14622 |
|  |  |  |  |  | 8 | group20_3871 | TGCTTTACCATAACCTTTATGAAAACA | CCTCTCCCCAATCTTTTTCAAAAATACCTTTTGA | ATTTAGCTGGACATTGGATTCTGATTA | - | 173 | gi|38230482 | 13137 | 13035 | 12991 |
| Influenza A MP Segment | [11320](http://www.ncbi.nlm.nih.gov/Taxonomy/Browser/wwwtax.cgi?id=11320) | 3964 | ~50 | N | 352 | 19 | CATTCCATGGAGCTAAGGAGGTT | CTCAACCGGTGCACTTGCCAGC | TGCTCACAAGTGGCACACACTA | + | 136 | gi|9438002 | 323 | 357 | 437 |
|  |  |  |  | N | 337 | 276 | CTGCTCACAAGTGGCACACA | AGGCCAAAAGCCACTTCCGTAGTCACC | AGCTACTCAACCGGTGCACTT | - | 108 | gi|95116891 | 440 | 411 | 352 |
|  |  |  |  | N | 342 | 467 | GTCTATGAGACCGATGCTGTGAGT | CAATCTGCTCACAAGTGGCACACACTAGG | CTAAGGAGGTTGCACTCAGCTACTC | - | 156 | gi|115278119 | 492 | 460 | 360 |
|  |  |  |  | N | 283 | 334 | TTCACTTGATCCCGCCATCT | CGATGCTGCGAATCTGCAATCTGCT | TGGCCTAGTGTGTGCCACTT | - | 160 | gi|93212846 | 572 | 455 | 432 |
| Influenza B MP Segment | [11520](http://www.ncbi.nlm.nih.gov/Taxonomy/Browser/wwwtax.cgi?id=11520) | 237 | 4 |  | 200 | group1_29 | AGGCCTGATTCTAGCTGAGAGAAA | TCATGGTCATGTACCTGAACCCTGGAAATT | GCGTTCCTAGTTTTACTTGCATTG | + | 158 | gi|90092215 | 306 | 407 | 440 |
|  |  |  |  |  | 4 | group3_38 | TGCGAGTGCGATGCTTGT | CTCGCATAAAGCACAGAGCGTTCCTAGTTTTACT | CCTAAACCCTGAAAACTATTCAATGC | - | 81 | gi|94481581 | 459 | 423 | 396 |
|  |  |  |  |  | 211 | group16_15 | TAGCTGAGAGAAAAATGAGAAGATGTG | TCTCATGGTCATGTACCTGAATCCTGGAAATT | GCGTTCCTAGTTTTACTTGCATTGA | + | 147 | gi|66710570 | 263 | 351 | 385 |
|  |  |  |  |  | 150 | group23_36 | GCACAGAGCGTTCCTAGTTTTACTT | CCAGGATTCAGGTACATGACCATGAGACAATACA | AGCTGAGAGAAAAATGAGAAGATGTGT | - | 153 | gi|50300210 | 422 | 374 | 294 |
| H1 Influenza A HA Segment | multiple nodes | 634 | 19 | N | 388 | 1266 | TGCTTATGTCTCTGTAGTGTCTTCACA | CTACTACTGGACTCTGCTGGAACCCGGG | AAAGCCTCTACTCAGTGCGAAAG | + | 196 | gi|94960083 | 655 | 751 | 828 |
|  |  |  |  | N | 388 | 1431 | AGGATGGTTACAGGACTAAGGAACA | CCATCCATTCAATCCAGAGGTTTGTTTGG | CCTTGTTTGTAATCCCGTTAATGG | + | 199 | gi|94960083 | 1004 | 1031 | 1179 |
|  |  |  |  | N | 31 | 1879 | TCCCCAAGGCAAGTTCATG | CCCTTATGCTGGAGCAAACAGCTTCTATAGAAATTT | GGATGGTGAATGCCCCATAG | + | 196 | gi|90101875 | 401 | 459 | 577 |
|  |  |  |  | N | 66 | 1881 | CCAAGATATGCCTTCGCAATG | TCACAATTGGAGAATGTCCAAAATACGTCAAAA | CCTGTAGCCATTCTCAATTTTGTG | + | 200 | gi|90101875 | 802 | 944 | 978 |
|  |  |  |  | N | 448 | 334 | CAGAGGTTTGTTTGGAGCCATT | CACAAAATGCCATTAACGGGATTACAAACAAGGT | CAGCTGTGAATTGAGTGTTCATTTT | + | 200 | gi|94960083 | 1045 | 1170 | 1220 |
|  |  |  |  | N | 392 | 4290 | GCGAAATACCCTGGGTAACATG | CGGCAACGCTGCAATTACCCAATTGTA | GTCAACCTACTTGAGGACAGTCACA | - | 200 | gi|94960083 | 336 | 219 | 158 |
|  |  |  |  | N | 8 | 4758 | GAAGACTACTGTTCAAGGCACCATAA | TGGTGCTATTAAGTTCCCAGTTGCCTCAAA | TATTATTGGACACTATTGGATCAAGGA | - | 187 | gi|113531188 | 915 | 793 | 754 |
|  |  |  |  | N | 421 | 4269 | CAAATGTCTAGAAACCCATCATCAAC | CACCTTGTTTGTAATCCCGTTAATGGCATTTTG | GAATGAGCAAGGATCTGGCTATG | - | 195 | gi|94960083 | 1298 | 1172 | 1129 |
|  |  |  |  | N | 300 | 256 | CTTTCGCACTGAGTAGAGGCTTT | CACCCAGTCACAATAGGAGAGTGTCCAAAGTATGTC | GTTCCTTAGTCCTGTAACCATCCTTAA | + | 200 | gi|94960083 | 828 | 953 | 1001 |
|  |  |  |  | N | 216 | 47 | GGTGTTTTGAATTCTACCACAAGTGT | TCTATCAGATTCTGGCGATCTACTCAACTGTCG | GGAGACTAAAAGCACCAGTGAACTG | + | 200 | gi|3831770 | 1471 | 1612 | 1646 |
|  |  |  |  | N | 374 | 897 | AAGGTGAACTCTGTTATCGAGAAAATG | CTCAATTCACAGCTGTGGGTAAAGAATTCAACAAAT | TTCTTCACATTTGAGTCATGGAAATC | + | 200 | gi|89782381 | 1202 | 1233 | 1376 |
| H2 Influenza A HA Segment | multiple nodes | 106 | 6 |  | 19 | group55_520 | GACTCCTCTTCATACTTGGGATAATCA | TGCATTGTCTCTCAGTTGCATTCTGACTTTATCAT | GTTCTAATGGAAAATGAGAGGACACTT | - | 200 | gi|408672 | 1534 | 1419 | 1361 |
|  |  |  |  |  | 16 | group76_813 | GACTCCTCTTCATACTTGGGATAATCA | CGCATTGTCCCTCAGTTGCATTCTGACTTTAT | GTTCTAATGGAAAATGAGAGGACACTT | - | 200 | gi|78097886 | 1534 | 1422 | 1361 |
|  |  |  |  |  | 57 | group235_972 | TTTTCTTTCTCCATTATGTAGGACCAT | CAATCCCCTAGTTCAAGTGGAGGGATTCC | CAATTCTAGAGCGGAATGTCACTGT | - | 199 | gi|9802416 | 290 | 201 | 118 |
|  |  |  |  |  | 26 | group246_124 | TTTTGAGAGCACTGGTAATTTAATTGC | CATTGCCTTTTCACAACATTCACCCATTG | TTACATATTTGGGACACTCACCTATTG | + | 200 | gi|78058794 | 804 | 947 | 977 |
|  |  |  |  |  | 44 | group469_481 | TTCTTTGTCAGCCAGACCATGT | CTTTCTCGAAATGTGTCACGCTGCTGA | TTGTGTTATCCAGGCAGCTTCA | - | 191 | gi|78097490 | 521 | 401 | 352 |
|  |  |  |  |  | 29 | group483_702 | GAGTTCGGCATTATATGTCCATACATC | ACTTTGTTAGTTATCCCATCAATTGCCTTTTGAGTG | CAGGATATGCAGCAGACAAAGAA | - | 200 | gi|78097192 | 1330 | 1182 | 1157 |
|  |  |  | OR Alternate MSC soln with shorter amplicons. | | | | |  |  |  |  |  |  |  |  |
|  |  |  | 7 |  | 13 | group43_592 | TGATAATCATATGCAGATCCTGCATT | CCCAGCTATCATGATTGCCAATGACAGG | GATCAAAGGAGTAAAATTGAGCAACAT | - | 160 | gi|134026367 | 1699 | 1637 | 1565 |
|  |  |  |  |  | 2 | group91_781 | ATCCATACCAACCATCAACCATT | CAATCCTGTTGCTAAGACCAATCTTTCCGATTTT | CATAGGTGAGTGCCCCAAATATG | - | 155 | gi|408518 | 1111 | 1003 | 979 |
|  |  |  |  |  | 31 | group135_993 | AAACCCCCATATTGCTCAATTTC | CCTGACACTATTCATGCATTCATCATCACATTTATG | AAGGAAATAGGGAACGGATGCT | - | 160 | gi|78058760 | 1590 | 1486 | 1453 |
|  |  |  |  |  | 52 | group287_636 | AGTTACTGAATTCTTTCCCAACAGCTT | TCTGCTGCATACCCTGATCCCTGGTC | TGGTATGGATACCATCACAGCAA | - | 157 | gi|94481531 | 1211 | 1105 | 1081 |
|  |  |  |  |  | 27 | group387_891 | TGCATTCTGACTTTGTCGTACAGAT | CCATCAAAACTAGGAGTTCGGCATTATATGTCCAT | AAATAAAAAGATGGAAGATGGGTTTC | - | 135 | gi|78058684 | 1412 | 1335 | 1302 |
|  |  |  |  |  | 38 | group486_1034 | TCTCAAAATTTATGGTGTCCCACAT | TCGACCTTTTGTTCAATGTTGATGTGCCTAC | CTGTACCAGAATGTGGGAACCTATG | - | 160 | gi|9802416 | 765 | 660 | 630 |
|  |  |  |  |  | 14 | group520_54 | GGGTTATACCATGGACAAACAAACA | TGCCAACAATTCCACAGAAAAAGTCGACA | GTGACGTTTCGCTCCAAGATT | + | 150 | gi|49357173 | 12 | 111 | 141 |
| H3 Influenza A HA segment | multiple nodes | 1590 | 23 | N | 227 | 4656 | TTGATGCCTGAAACCATACCAA | TGCTTGACATATTTGGGACATGCCCC | ATGACAAGCCCTTTCAGAATGTAAAC | - | 200 | gi|52078172 | 1095 | 955 | 917 |
|  | | |  | N | 216 | 6651 | ACGTATTTCTCGAGGTCCTGAATT | TGCTTGTCCTGTGCCCTCAGAATTTTGA | ATAGACGGTTGGTACGGTTTCAG | - | 200 | gi|94481543 | 1263 | 1113 | 1087 |
|  |  |  |  | N | 466 | 1574 | GACTCAGAAATGAACAAACTGTTTGAA | ACCACAAATGTGACAATGCCTGCATAGG | AGCTCAACACCTTTGATCTGGAA | + | 200 | gi|99093049 | 1399 | 1487 | 1576 |
|  |  |  |  | N | 408 | 2467 | GGGTCAATCAGAAATGGAACTTATG | TGGATCCTATGGATTTCCTTTGCCATATCATG | TTTTTGGCAGGCCCACAT | + | 189 | gi|99093049 | 1513 | 1618 | 1684 |
|  |  |  |  | N | 408 | 5741 | CCTCAGAATTTTGATGCCTGAAA | CCTGTTGCCAATTTCAGAGTGTTTTGCTTAACATA | TCAAAATGTAAACAGGATCACATATGG | - | 197 | gi|99093049 | 1134 | 1000 | 960 |
|  |  |  |  | N | 393 | 3928 | TGAAATGGTTTGTCATTGGGAAT | CCCGAGGAGCAATTAGATTCCCTGTGC | GGACAATAGTAAAACCGGGAGACATA | - | 184 | gi|94481527 | 910 | 788 | 749 |
|  |  |  |  | N | 357 | 6138 | TGATGCCTGAAACCGTACCA | CCTGTTGCCAATTTCAGAGTGTTTTGCTTAACATAT | ATGACAAACCATTTCAAAATGTAAACA | - | 199 | gi|94960121 | 1103 | 976 | 924 |
|  |  |  |  | N | 413 | 3162 | GTTACTTCAAAATACGAAGTGGGAAAA | CCCAATGACAAACCATTTCAAAATGTAAACAGG | GCATCCCTGTTGCCAATTTC | + | 195 | gi|94960121 | 822 | 920 | 997 |
|  |  |  |  | N | 254 | 3722 | AATGCAAATGTTGCACCTAATGTT | TCAACACCTTTGATCTGAAACCGGTTGTTT | CCATGATGTATACAGAGACGAAGCA | - | 190 | gi|94481543 | 1675 | 1536 | 1509 |
|  |  |  |  | N | 255 | 3895 | TCAAATGCAAATGTTGCACCTA | CAACACCTTTGATCTGGAACCGGTTGTT | CCATGATGTATACAGAGACGAAGCA | - | 193 | gi|99093049 | 1710 | 1567 | 1539 |
|  |  |  |  | N | 350 | 4159 | ATTCCCTCCCAACCATTTTCTAT | CCCCATATGTGATCCTGTTTACATTTTGAAATGG | GAATGCATCACTCCAAATGGAA | - | 200 | gi|94960121 | 1070 | 932 | 893 |
|  |  |  |  | N | 237 | 1934 | TGAAGACTATCATTGCTTTGAGCTACA | CAAACGGAACGCTAGTGAAAACAATCACG | TACCTGTTGAGGAACTCTGAACCA | + | 195 | gi|94960045 | 10 | 118 | 181 |
|  |  |  |  | N | 476 | 5250 | CTGAGCGACTCCAGTCCAATT | AGGTCCCATTCCTTATTTTGGAAGCCATCAC | ATGCTCTATTGGGAGACCCTCAT | - | 194 | gi|94960121 | 431 | 282 | 258 |
|  |  |  |  | N | 60 | 1416 | AATTCTGAGGGCACAGGACAA | AGAAATTCCATCAAATCGAAAAGGAATTCTCAGAA | AACGTATTTCTCGAGGTCCTGAAT | + | 171 | gi|94481543 | 1117 | 1217 | 1264 |
| H5 Influenza A HA segment | multiple nodes | 1137 | 6 |  | 359 | group6_61 | ATGTAAGACCATTCCGGCACAT | CTTCCCGTTGTGTGTCTTTTCCAGTATGTCTTG | CAGAGCAGGTTGACACAATAATGG | - | 199 | gi|99030939 | 291 | 164 | 114 |
|  |  |  |  |  | 394 | group34_81 | CATTTTCCATGAGAACCAGAAGTTC | CTGCCTCAAACTGAGTGTTCATTTTGTCAATGATT | AGAATCCACTCAAAAGGCAATAGATG | - | 200 | gi|4240451 | 1323 | 1196 | 1148 |
|  |  |  |  |  | 326 | group39_85 | AAGTCCAGACATCTAGAAATCCGTCTT | CGGCCTCAAACTGAGTGTTCATTTTGTCAAT | GAGTGGATACGCTGCAGACAAA | - | 188 | gi|57208068 | 1265 | 1177 | 1104 |
|  |  |  |  |  | 18 | group48_114 | CAGTGTAGCTGGGTGGCTTCTT | CCTGTGTTACCCTGGAAACTTCGACAATTATGA | ATCTGAACTCTCTCAAAGTGATTTGTG | + | 183 | gi|134122731 | 213 | 312 | 369 |
|  |  |  |  |  | 345 | group110_66 | ATGTAAGACCATTCCGGCACAT | CTTCCCATTGTGTGTCTTTTCCAGTATGTCTTGG | CAGAGCAGGTTGACACAATAATGG | - | 199 | gi|66820231 | 263 | 135 | 86 |
|  |  |  |  |  | 380 | group111_67 | CCGTTTCTTACACTTTCCATACATTC | CCCTAAGCTGTAGTCGAACCTTGTCGTAAAGGTTC | TGGACTTATAATGCTGAACTTCTGGTT | - | 191 | gi|89994622 | 1465 | 1374 | 1300 |
| HIV 1 | [11676](http://www.ncbi.nlm.nih.gov/Taxonomy/Browser/wwwtax.cgi?id=11676) | 1107 | 11 |  | 207 | group4_80694 | GTCTATTATTCTTTCCCCTGCACTGT | CCCCTTTTCTTTTAAAATTGTGAATGAATACTGCCA | CAGGAATTTGGAATTCCCTACAA | - | 198 | gi|57338562 | 4208 | 4160 | 4036 |
|  |  |  |  |  | 15 | group6_17063 | AGTGAGAAGCAGTGTGTGCTCAT | TCCGACGCAACGGGCTCG | TGTGAGTTCCTCTCGCCTCTTAG | + | 200 | gi|51599138 | 579 | 717 | 754 |
|  |  |  |  |  | 3 | group8_11422 | CAGCGGCAGCAGCTGATA | CGCTGACACCCAGAACTTTAAATGCTTGG | GGGTGTTGCCCCTTCTGA | + | 185 | gi|67866567 | 625 | 703 | 792 |
|  |  |  |  |  | 3 | group8_15194 | AGAAATCACTCTTGGAATTGGATCA | AGCAAGAGTTAGGCAGGGATATTCTCCCCTCTC | TGTTGGGATAAGGGTCTGCAA | + | 200 | gi|67866567 | 7664 | 7809 | 7843 |
|  |  |  |  |  | 3 | group8_66420 | CAAGTCTCATTCCAAGGCACAGT | AGCATATAGTTTTTCCTGAGCAGCCCCATAGAC | AGATACCTTAGGGATCAGCAAATCC | - | 89 | gi|67866567 | 7510 | 7472 | 7444 |
|  |  |  |  |  | 18 | group13_32917 | CCTGGTATCTAGAGATCCCTCAGATC | TCCGACGCAACGGGCTCG | CCACCGCCAGCAAATTTAC | + | 194 | gi|51599129 | 612 | 719 | 787 |
|  |  |  |  |  | 191 | group14_2030 | CTGACGGTACAGGCCAGACA | CAGCTCCAGGCAAGAGTCCTGGCT | TTCCAGAGCAGCCCCAAA | + | 193 | gi|8886632 | 7100 | 7214 | 7275 |
|  |  |  |  |  | 222 | group21_12566 | TCCAGTCAGACCTCAGGTACCTTT | CACAAGGCTACTTCCCTGATTGGCAGAA | CCCTGGCCCTGGTGTGTA | + | 193 | gi|66864700 | 9001 | 9147 | 9176 |
|  |  |  |  |  | 309 | group22_75711 | CACCTGCCATCTGTTTTCCAT | TGGTCCTTTCCAAATAGGGTCTCTGCTGTC | AAATTCAAAATTTTCGGGTTTATTACA | - | 177 | gi|57338562 | 4439 | 4312 | 4283 |
|  |  |  |  |  | 8 | group23_47661 | ACCTGCCATCTGTTTTCCATAATC | TCCTTTCCAAATAGGGTCTCTGCTGTCTCTGT | TTTAAAAATTCAAAATTTTCGGGTCTA | - | 181 | gi|51599138 | 5081 | 4953 | 4924 |
|  |  |  |  |  | 213 | group27_16329 | CTGTACCAGTAAAATTAAAGCCAGGAA | CCCAAAAGTTAAACAATGGCCATTGACAGA | TGTATGGATTTTCAGGCCCAAT | + | 150 | gi|9629357 | 2115 | 2149 | 2243 |
|  |  |  |  |  | 364 | group29_17149 | GACTCTGGTAACTAGAGATCCCTCAGA | CTCTAGCAGTGGCGCCCGAACAG | CGAGTCCTGCGTCGAGAGA | + | 144 | gi|9629357 | 123 | 173 | 228 |
|  |  |  |  |  | 252 | group37_40960 | GCTATGTCACTTCCCCTTGGTT | TGCATGGCTGCTTGATGTCCCC | AGAAGGAGCCACCCCACAA | - | 183 | gi|9629357 | 1024 | 910 | 863 |
| **Bacteria** |  |  |  |  |  |  |  |  |  |  |  |  |  |  |  |
| Haemophilus influenzae | [727](http://www.ncbi.nlm.nih.gov/Taxonomy/Browser/wwwtax.cgi?id=727) | 15 | 1 |  | 15 | group3_1204 | CCATGGAAACTGACCCAATAGATA | TGCAACAGCAAGCCCAGCACG | TGTAAAAGAAAAATTATTGCGTTTTGC | + | 95 | gi|68248551 | 827542 | 827588 | 827610 |
|  |  |  |  |  | 15 | group3_103 | CAGGCTTTGAGCGTTTAAATAAATATG | TCCTCGCAATGCAGCGGCAG | GGATCAAGCTGGCGTAAAGAG | - | 100 | gi|68248551 | 1200252 | 1200201 | 1200179 |
|  |  |  |  |  | 15 | group3_5670 | ATCTTGTTTGGGTTCGCAAAAA | CACCGCCACGAGTAACGAGTACATTTTCAG | GAATTATGCGGGCGTTTCA | - | 100 | gi|68248551 | 562188 | 562130 | 562110 |
|  |  |  |  |  | 15 | group3_181 | GGACATCAGCCCAGCCTTATTA | TCACCTTTAGCTGCTTTAAATGCTTCAGCCA | CAAGAAGATATTGTTATTCCAAATGCA | + | 95 | gi|68248551 | 1790842 | 1790878 | 1790910 |
|  |  |  |  |  | 15 | group3_1135 | CTCTTGGCGGTGCGTTACT | TTTTGTTTCCGCGTGCCTTTGCTAAAT | CAAGCCACAAATATCGGCTGTA | - | 100 | gi|68248551 | 200164 | 200106 | 200083 |
| Mycobacterium tuberculosis | [1773](http://www.ncbi.nlm.nih.gov/Taxonomy/Browser/wwwtax.cgi?id=1773) | 8 | 1 |  | 8 | group4_1 | GTTGTATGCGCACGTCCTTT | TTTCGCACCGCTCCGATACCG | CATGCCACGTTTTGCTCTTC | + | 300 | gi|148659757 | 3498341 | 3498373 | 3498621 |
|  |  |  |  |  | 8 | group4_2 | CGGACTATAGATCTCACGCAATATAGC | CGATCATTTCCACGAGCTACGATGCTC | CTTGCTGGCTGGGCAACT | + | 79 | gi|148659757 | 4199128 | 4199161 | 4199189 |
|  |  |  |  |  | 8 | group4_9 | CCACACACCAGGAGCCAGTA | CGCTCACAACTCGCAGCGTAGTTTGA | GCTCGCTAACCGCCAACT | - | 147 | gi|148659757 | 2229544 | 2229440 | 2229417 |
|  |  |  |  |  | 8 | group4_10 | GGAATCGAGGTGCTTGTCATC | CCTCTCGGATCGTTATCGCATGCC | GTACCCGGAAGAGCAAAACG | - | 71 | gi|148659757 | 3498664 | 3498635 | 3498614 |
| Staphylococcus aureus | [1280](http://www.ncbi.nlm.nih.gov/Taxonomy/Browser/wwwtax.cgi?id=1280) | 12 | 1 |  | 12 | group6_100 | CCTTACATTGATGCTGAGCGTTT | CCCTAACTCCCCACAAATCTGGAACGATACT | ACATCCAATATAGGACGTGGCTTT | - | 231 | gi|151220212 | 2613807 | 2613624 | 2613599 |
|  |  |  |  |  | 12 | group6_12 | TGTTGGAGGATTACGCCCTTT | AATCTTACGGCCTTCAACAACTAGCTCATACCAA | TCAGTGTGTTTTGATGCACCAA | + | 101 | gi|151220212 | 1607338 | 1607382 | 1607417 |
|  |  |  |  |  | 12 | group6_112 | CCATATGCACGTGGTTCCTTAC | TTCGTACTCTTGGTGCGCATAGTCCATTTT | CCTTTAGCAATGCCGGTGTT | - | 83 | gi|151220212 | 492589 | 492549 | 492528 |
|  |  |  |  |  | 12 | group6_42 | AGGTATCGACAATACTTATCGGAGACA | CCAGTAACACTAATTCCGGATACGGCAACAAATA | GGTGTAGAAGTAAATCCAATTGACACA | - | 91 | gi|151220212 | 992403 | 992367 | 992339 |
|  |  |  |  |  | 12 | group6_161 | TGAATTTTGCGAAACCAGTAATAAGT | TGCGCCTAGCGTCGTACCACGA | GTCGGCGGTGGACCTTAC | - | 94 | gi|151220212 | 2381785 | 2381743 | 2381717 |
|  |  |  |  |  | 12 | group6_163 | GTATTAAAGTAACCTGCGCCTATTGTG | CACCACTGTTACTTACTGATGCAACTAATTTCGGTG | TCTATATAAGGAGCGAACAGCTATGTG | - | 171 | gi|151220212 | 882131 | 882088 | 881987 |
| Ehrlichia genus | [943](http://www.ncbi.nlm.nih.gov/Taxonomy/Browser/wwwtax.cgi?id=943) | 6 | 3 |  | 1 | group1_95 | CAGGGTACCATTGTCTTTCAGGTT | CCCTGCAAAACTGCTTGCATAAGTAGGGTC | AATACTCACAGCAATTTTACACGATCA | + | 154 | gi|73666633 | 1282967 | 1283061 | 1283094 |
|  |  |  |  |  | 1 | group1_218 | TACTACAGTGCACATGACCCTATATCG | CCATCTGGCCAATCTAATAAATCCAGTGCA | GCTAGTGCAAAACATCAAGCATATG | - | 95 | gi|73666633 | 1143211 | 1143169 | 1143143 |
|  |  |  |  |  | 2 | group3_121 | CATCCGAGGTTCTCTTATCTCACTAA | ACGTGCAATACGCCTAACACGTCTTTCC | ACCATTTTATGCAAGGAGTGCAA | - | 83 | gi|88657561 | 227652 | 227620 | 227595 |
|  |  |  |  |  | 2 | group3_100 | CTTGTGTAACTTCTGATACCTCCTGAA | CCACTGCGTTTAGTCGTACCCTTGACTAATTGC | AGAACTAGAATTCGTAGGATGCATCA | - | 133 | gi|88657561 | 428687 | 428643 | 428581 |
|  |  |  |  |  | 3 | group7_25 | AATCTACATGCGCATAATGCCTATT | CATACTCTACATGTGCTGTTGAAATGGTAATCCCTC | GAAGAGATTAAGTGGTGAAGGGAATAA | + | 143 | gi|58578664 | 1002290 | 1002323 | 1002406 |
|  |  |  |  |  | 3 | group7_272 | TACATCGTGAACATGACTTTCACGTA | CATTGCAGATCTTAACCCACCAATTAACTGGTGT | TTGAAAGGTCCAATCTCTGGAGTA | - | 159 | gi|58578664 | 1259131 | 1259024 | 1258998 |
| Ehrlichia chaffeensis | [945](http://www.ncbi.nlm.nih.gov/Taxonomy/Browser/wwwtax.cgi?id=945) | 2 | 1 |  | 2 | group1_12 | ACTGCAGATGCACCTGGTATAGG | AGGTTTACTTCCTTCTGTCTGCGACGCTAATGTA | AACCTTATTCTATACCAGGTGCATCTG | + | 142 | gi|88657561 | 658858 | 658995 | 659084 |
|  |  |  |  |  | 2 | group1_15 | CATCCGAGGTTCTCTTATCTCACTAAA | ACGTGCAATACGCCTAACACGTCTTTCC | ACCATTTTATGCAAGGAGTGCAA | - | 83 | gi|88657561 | 227651 | 227620 | 227595 |
| Ehrlichia ruminantium | [779](http://www.ncbi.nlm.nih.gov/Taxonomy/Browser/wwwtax.cgi?id=779) | 3 | 1 |  | 3 | group4_45 | ACCACATAAGATACCCATTACGTATCC | AGCTAAGACATTTGCGTTAACAATACCTGCGAAATT | TTATCAACACTACGAGAGGATTTTGCT | + | 129 | gi|58578664 | 411445 | 411481 | 411547 |
| Chlamydia trachomatis | [813](http://www.ncbi.nlm.nih.gov/Taxonomy/Browser/wwwtax.cgi?id=813) | 6 | 1 |  | 6 | group2_1027 | CCAAAGACATAGTCTTGGGATGAC | CCGGAAGGAGCTACCAGCCCACTT | AGATCGAGTTATCGGCACCATAG | - | 81 | gi|76788711 | 796510 | 796477 | 796453 |
|  |  |  |  |  | 6 | group2_102 | ATTCATGTCTTTCTCGAATCTTTTCA | CGCTCTCTTCCCTTTAGGAAGCCGACA | AGCGCAGTAGCTGGTGTTACG | + | 100 | gi|76788711 | 416368 | 416402 | 416447 |
|  |  |  |  |  | 6 | group2_1069 | GATACTGCTCATCCCAACGAACTA | CGGCATGCTGAGTCTTCTCAATACGATTTACTACA | TGCAACGTGTCATTCGAATTT | + | 153 | gi|76788711 | 524226 | 524253 | 524358 |
|  |  |  |  |  | 6 | group2_1148 | TACTCATGTCCTTCCCTCTCCTCTT | CGGACACCTACCTAGAAATTGCCCACACTC | AGTGAGGGTGAGGATAGGAAACAT | - | 83 | gi|76788711 | 26128 | 26097 | 26070 |
| Pseudomonas aeruginosa | [287](http://www.ncbi.nlm.nih.gov/Taxonomy/Browser/wwwtax.cgi?id=287) | 7 | 1 |  | 7 | group27_1068 | ATAGTGCTTCAAATAGCCATCACTTG | CGGAGCGTTCACGCCCCA | TCAGTCATCACCCGGTTTCTT | + | 86 | gi|152983466 | 1190214 | 1190251 | 1190279 |
|  |  |  |  |  | 7 | group27_1577 | TTCCCCTGTGGTTGGCTTT | CCAATTGATCCCACTTTTACCCACTTCATACC | AGTGTGGATTTCTATAGTGTGCAGAAG | + | 84 | gi|152983466 | 5151012 | 5151036 | 5151069 |
|  |  |  |  |  | 7 | group27_184 | TTTTTCCCGGGCCTAACC | TCCTATCTGTTTCGTGGCCAAGTGCC | GAGTGTTTTCATAATGTCGGCTCTAC | - | 94 | gi|152983466 | 3622388 | 3622341 | 3622312 |
|  |  |  |  |  | 7 | group27_2042 | TCCCATGACAGTCCCTCCTT | CGCATGTATTGCGCCTGCCATG | TGAACCCAAGGCTTTTCTACACT | + | 80 | gi|152983466 | 6418898 | 6418929 | 6418955 |
|  |  |  |  |  | 7 | group27_2246 | GGGACGCCGGTCTACTCTT | TTGTCAGGTCGCCCGATTCTCCTTC | GACAGGCCCCTTGTCCAA | - | 81 | gi|152983466 | 4063846 | 4063804 | 4063784 |
|  |  |  |  |  | 7 | group27_4009 | CTCCGATAAGAATAATTCGCAACTC | TTGCTTTCTATCCCCGTTTGTTTCCCA | CGCTAACCAAGCGCTGATT | - | 129 | gi|152983466 | 1564687 | 1564603 | 1564583 |
| Escherichia coli | [562](http://www.ncbi.nlm.nih.gov/Taxonomy/Browser/wwwtax.cgi?id=562) | 42 | 1 |  | 42 | 1 | TTATCTGCTTAGCGTGCTGGAA | TCTGTTATCGCCTCCGCTGTCGG | CGCCCGACATAACGTGAAG | + | 228 | gi|117622295 | 3220907 | 3221034 | 3221116 |
|  |  |  | OR Alternate MSC soln with shorter amplicons. | | | | |  |  |  |  |  |  |  |  |
|  |  |  | 2 |  | 37 | group6_1790 | CGCATGTCGTTACGCCATAA | CAATCACCACAGATGGAACAGGTACGGC | GAGCGGGCATACAGCAAAA | + | 97 | gi|75186776 | 3146519 | 3146553 | 3146615 |
|  |  |  |  |  | 16 | group11_1327 | CAATACTTTGAAACCCACAGCTGTA | TTTCAGACCAGCACGTGAACGTATCTTCA | ACCTTTGAGTGTCTCCCCACAT | - | 106 | gi|89106884 | 4007415 | 4007341 | 4007287 |
|  |  |  |  | N | 35 | 573 | GTAACGACAATGGGCGAATTACT | CGCCTCGATTATGTTCTTTGCGCC | GCTGAGGTGGCGAACGAT | - | 146 | gi|49175990 | 361592 | 361565 | 361469 |
|  |  |  |  | N | 34 | 1762 | GTAACACCCACGGAGGTAATACATC | TCTGGCACCAGTGGTTACAGTTCTCTTAATTATCGT | GCTGTCACCACTCCGACTGT | - | 110 | gi|49175990 | 1587529 | 1587492 | 1587444 |
|  |  |  |  | N | 35 | 1454 | GGCTTACTGAACAAATATCCCCTTT | CGATATATAAAAATCGATCGTCCACATGCAGAGA | GCACGATTGCAAGCCTCAT | - | 193 | gi|117622295 | 316244 | 316127 | 316076 |
|  |  |  |  | N | 35 | 33 | GTAACGACAATGGGCGAATTACT | CGCCTCGATTATGTTCTTTGCGCC | GCTGAGGTGGCGAACGAT | - | 146 | gi|49175990 | 361592 | 361565 | 361469 |
|  |  |  |  | N | 32 | 454 | GCCTCCGGGCGATAAACT | TGGGCCTTCCGGATCCACAACA | GCTTACGTCTACACGCTGGCTAA | + | 158 | gi|15829254 | 2117547 | 2117567 | 2117682 |
| Neisseria meningitidis | Top of Form  [487](http://www.ncbi.nlm.nih.gov/Taxonomy/Browser/wwwtax.cgi?id=487) | 3 | 1 |  | 3 | group3_120 | CCCACCGCACCCATAGAC | CCAAGGCAACAGTTCGTACTACATTGCCA | AACGGCAAATGTGCAGGAT | - | 171 | gi|121633901 | 67981 | 67867 | 67828 |
|  |  |  |  |  | 3 | group3_36 | GGACAAGTGACTGTTCAGTCCTATTT | CGGACAGATTGTATGCCTACCAATCCGG | GCCCAATCCGCCATTTAA | + | 189 | gi|121633901 | 557588 | 557724 | 557759 |
|  |  |  |  |  | 3 | group3_33 | TGCGGTACAAGGATGATGTTG | CTTCCGTGTCAGGATGTCTGCCTGATACA | TGAACGCATACGTGGCAAATAC | + | 129 | gi|121633901 | 2114744 | 2114773 | 2114851 |
|  |  |  |  |  | 3 | group3_39 | GGCCTATGATGGCGATGTC | TGCTCAAATCTATTTCAAACGGTGCGTAACGT | GCAACGTATACCGGGCAAA | + | 104 | gi|77358697 | 1099942 | 1099972 | 1100027 |
|  |  |  |  |  | 3 | group3_48 | GGGAAGATTTCCTCATGGTACTTC | ACATCGGATCCGCCGTCCAAAC | GAGCAGATTCCGGCAGAGTT | - | 118 | gi|15793034 | 1316951 | 1316878 | 1316857 |
|  |  |  |  |  | 3 | group3_88 | ATGTACCTTAGCCGTTCGGCTAT | CATGCGTTCTCAAAGCTGAACGCG | GAATCTTTCAAGATTCCGCACAGT | - | 127 | gi|15793034 | 1742021 | 1741993 | 1741917 |

No signatures are predicted to have any false positives, except for two of the Newcastle disease virus signatures indicated with ^1^, which are predicted to detect Goose paramyxovirus. Goose paramyxovirus is not currently classified under the same taxonomy node as Newcastle disease virus and pigeon paramyxovirus.
